# Associations with photoreceptor thickness measures in the UK Biobank

**DOI:** 10.1038/s41598-019-55484-1

**Published:** 2019-12-19

**Authors:** Sharon Y. L. Chua, Baljean Dhillon, Tariq Aslam, Konstantinos Balaskas, Qi Yang, Pearse A. Keane, Adnan Tufail, Charles Reisman, Paul J. Foster, Praveen J. Patel, UK Biobank Eye and Vision Consortium, Prof. Paul Bishop, Prof. Sarah A. Barman, Prof. Jenny H. Barrett, Mr. Peter Blows, Dr. Catey Bunce, Dr. Roxana O. Carare, Prof. Usha Chakravarthy, Dr. Michelle Chan, Prof. David P. Crabb, Mrs Philippa M. Cumberland, Dr. Alexander Day, Dr. Parul Desai, Prof. Cathie Sudlow, Prof. Andrew D. Dick, Dr. Cathy Egan, Prof. Sarah Ennis, Dr. Marcus Fruttiger, Dr. John E. J. Gallacher, Prof. David F. Garway-Heath, Dr. Jane Gibson, Mr. Dan Gore, Prof. Jeremy A. Guggenheim, Prof. Chris J. Hammond, Prof. Alison Hardcastle, Prof. Simon P. Harding, Dr. Ruth E. Hogg, Dr. Pirro Hysi, Prof. Sir Peng T. Khaw, Dr. Anthony P. Khawaja, Dr. Gerassimos Lascaratos, Prof. Andrew J. Lotery, Dr. Tom Macgillivray, Dr. Sarah Mackie, Prof. Keith Martin, Ms. Michelle Mcgaughey, Dr. Bernadette Mcguinness, Dr. Gareth J. Mckay, Mr. Martin Mckibbin, Dr. Danny Mitry, Prof. Tony Moore, Prof. James E. Morgan, Ms. Zaynah A. Muthy, Mr. Eoin O’Sullivan, Dr. Chris G. Owen, Mr. Euan Paterson, Dr. Tunde Peto, Dr. Axel Petzold, Prof. Jugnoo S. Rahi, Dr. Alicja R. Rudnicka, Dr. Jay Self, Prof. Sobha Sivaprasad, Mr. David Steel, Mrs Irene Stratton, Dr. Nicholas Strouthidis, Dr. Caroline Thaung, Dr. Dhanes Thomas, Prof. Emanuele Trucco, Dr. Veronique Vitart, Prof. Stephen A. Vernon, Dr. Ananth C. Viswanathan, Dr. Cathy Williams, Dr. Katie Williams, Prof. Jayne V. Woodside, Dr. Max M. Yates, Dr. Jennifer Yip, Dr. Yalin Zheng, Dr. Robyn Tapp

**Affiliations:** 1 NIHR Biomedical Research Centre, Moorfields Eye Hospital NHS Foundation Trust and UCL Institute of Ophthalmology, London, United Kingdom; 2 Centre for Clinical Brain Sciences, School of Clinical Sciences, University of Edinburgh, Edinburgh, UK; 3 NHS Lothian Princess Alexandra Eye Pavilion, Edinburgh, UK; 4 Faculty of Biology, Medicine and Health, School of Pharmacy and Optometry, The University of Manchester, Manchester, UK; 5 Manchester Royal Eye Hospital, NHS Central Manchester University Hospitals, Manchester, United Kingdom; 6 School of Biological Sciences, University of Manchester, Manchester, UK; 7 Topcon Healthcare Solutions, Oakland, New Jersey United States of America; 9 Manchester University, Manchester, United Kingdom; 10 Kingston University, London, United Kingdom; 11 University of Leeds, Yorkshire, United Kingdom; 12 King’s College London, London, United Kingdom; 13 University of Southampton, Southampton, United Kingdom; 14 Queens University Belfast, Belfast, Ireland; 15 City, University of London, London, United Kingdom; 16 UCL Great Ormond Street Institute of Child Health, London, United Kingdom; 17 University of Edinburgh, Scotland, United Kingdom; 18 University of Bristol, Bristol, United Kingdom; 19 University of Oxford, Oxford, United Kingdom; 20 Cardiff University, Wales, United Kingdom; 21 University of Liverpool, London, United Kingdom; 22 University of Cambridge, Cambridge, United Kingdom; 23 Leeds Teaching Hospitals NHS Trust, Yorkshire, United Kingdom; 24 King’s College Hospital NHS Foundation Trust, London, United Kingdom; 25 St George’s, University of London, London, United Kingdom; 26 UCL Institute of Neurology, London, United Kingdom; 27 Newcastle University, Newcastle, United Kingdom; 28 Gloucestershire Hospitals NHS Foundation Trust, Gloucester, United Kingdom; 29 University of Dundee, Scotland, United Kingdom; 30 Nottingham University Hospitals NHS Trust, Nottingham, United Kingdom; 31 University of East Anglia, Norwich, United Kingdom

**Keywords:** Epidemiology, Risk factors

## Abstract

Spectral-domain OCT (SD-OCT) provides high resolution images enabling identification of individual retinal layers. We included 32,923 participants aged 40–69 years old from UK Biobank. Questionnaires, physical examination, and eye examination including SD-OCT imaging were performed. SD OCT measured photoreceptor layer thickness includes photoreceptor layer thickness: inner nuclear layer-retinal pigment epithelium (INL-RPE) and the specific sublayers of the photoreceptor: inner nuclear layer-external limiting membrane (INL-ELM); external limiting membrane-inner segment outer segment (ELM-ISOS); and inner segment outer segment-retinal pigment epithelium (ISOS-RPE). In multivariate regression models, the total average INL-RPE was observed to be thinner in older aged, females, Black ethnicity, smokers, participants with higher systolic blood pressure, more negative refractive error, lower IOPcc and lower corneal hysteresis. The overall INL-ELM, ELM-ISOS and ISOS-RPE thickness was significantly associated with sex and race. Total average of INL-ELM thickness was additionally associated with age and refractive error, while ELM-ISOS was additionally associated with age, smoking status, SBP and refractive error; and ISOS-RPE was additionally associated with smoking status, IOPcc and corneal hysteresis. Hence, we found novel associations of ethnicity, smoking, systolic blood pressure, refraction, IOP_cc_ and corneal hysteresis with photoreceptor thickness.

## Introduction

Optical coherence tomography (OCT) imaging has transformed our understanding of diseases affecting the vitreous, retina and choroid. It is a non-invasive, *in vivo* imaging modality which measures the optical reflectivity of the tissue of interest. With an axial resolution of approximately 5 µm, OCT imaging enables visualization and accurate measurement of retinal layers from the retinal nerve fibre layer through to layers relating to photoreceptors. Modern “segmentation” software algorithms make it possible to measure retinal layer thicknesses using changes in optical reflectivity to detect boundaries between retinal layers *in vivo*^1,2^. Though there is good agreement regarding the correlation between inner retinal boundaries and histology, the histological correspondence of some of the outer retinal boundaries relating to components of the photoreceptors is still a matter of debate^3–5^.

Photoreceptors play a central role in vision function and are responsible for phototransduction^6^. Since OCT imaging provides quantitative information on specific retinal layers, ocular diseases which affect the photoreceptors may be detected at an early stage, monitored for progression and plan preventive treatments using OCT imaging before the development of more severe disease^7–10^. Previous studies using ultra-high resolution OCT showed that the severity of photoreceptor loss is associated with decreased visual acuity in retinitis pigmentosa^7,11^. Other studies using spectral-domain (SD) OCT reported the thickness of outer nuclear layer (ONL) in the fovea is associated with visual acuity loss in ocular diseases such as central serous retinopathy^8,9^, polypoidal choroidal vasculopathy^12^, and epiretinal membrane^13,14^.

As OCT becomes more widely used to diagnose and monitor retinal pathologies, it is important to report normative data for photoreceptor layer thickness and identify major determinants of the photoreceptor layer thickness in populations. Although studies have distinguished the retinal layers of the photoreceptors in a normal population^11,15–19^, these studies had a small sample size of less than 300 and did not evaluate other ocular or systemic risk factors which may affect the retinal layers of the photoreceptors^15–17^. Examining ocular and systematic factors that affects photoreceptor layer thickness in normal aged eyes may allow the early detection of disease-related changes, and allowing earlier treatment of disease to reduce vision loss. We hypothesized that ocular and systemic factors are associated with photoreceptor layer thickness. This study aims to determine and describe the distribution of the photoreceptor layer among individuals with no ocular disease and to examine its association with demographic and risk factors in the UK Biobank data resource.

## Methods

UK Biobank is a large-scale multisite cohort study of UK residents aged 40–69 years who were registered with the National Health Service. The UK Biobank data resource was set up to allow detailed investigation of genetic and environmental determinants of major diseases of later life^20^. Detailed explanation of the study methodology has been published elsewhere^21,22^. Briefly, extensive baseline questionnaires, physical measurements, and biological samples were collected from participants at 22 assessment centres between 2006 and 2010^21^. Participants completed a touchscreen self-administered questionnaire on their general health and socioeconomic status. Ethnicity was categorised as White (English/Irish or other White background), Asian (Indian/Pakistani/Bangladeshi or another Asian background), Black (Caribbean, African, or other Black background), Chinese, Mixed (White and Black Caribbean or African, White and Asian, or other mixed background), or other ethnic group. The Townsend deprivation index was determined according to the participants’ postcodes at recruitment and the corresponding output areas from the preceding national census. The Townsend index was calculated based on the employment status, home and car ownership, and household condition. A higher and more positive index indicates a more deprived area. Smoking status was categorized as “Never”, “Previous”, or “Current”. Health examination included blood pressure and weight measurement^23^. Eye measurement including visual acuity (VA), autorefraction, Goldmann-corrected IOP (IOPg), cornea-corrected IOP (IOPcc) and corneal hysteresis were conducted in late 2009^23,24^. The study was approved by the North West Research Ethics Committee (reference number 06/MRE08/65). Informed written consent was obtained from the participants. It was conducted according to the tenets of the Declaration of Helsinki.

### Spectral-domain optical coherence tomography imaging protocol

Spectral-domain OCT imaging was performed using the Topcon 3D OCT 1000 Mk2 (Topcon Corp., Tokyo, Japan) after visual acuity, autorefraction and IOP measurements were collected. OCT images were obtained under mesopic conditions, without pupillary dilation, using the 3D macular volume scan (512 A-scans per B-scan; 128 horizontal B-scans in a 6 × 6-mm raster pattern)^25,26^.

### Analysis of macular thickness

All SD OCT images were stored as Topcon proprietary .fds image files on the UK Biobank supercomputers in Oxford, United Kingdom, with no prior analysis of macular thickness. The inner and outer retinal surfaces were segmented using the Topcon Advanced Boundary Segmentation (TABS) Algorithm (Version 1.6.1.1)^27^. Previous studies that used the TABS segmentation algorithm to segment specific retinal layers in the UK Biobank have been published^25,26^. Ko *et al*.^26^ evaluated the retinal pigment epithelium (RPE) which provides metabolic and functional support for the visual cells in the neural retinal and is located between the photoreceptors and choriocapillaris. Quality control measures during data collection included: 1) Image quality score; 2) The internal limiting membrane (ILM) indicator; 3) A validity count, and 4) Motion indicators. These quality control measured have been described previously^25,26^. In brief, the image quality score indicates the signal strength for the scan. The ILM indicator identifies blinks and scans that contain regions of severe signal attenuation or segmentation errors. The validity count indicator identifies scans with significant degree of clipping in the scan’s z-axis dimension. The motion indicator identifies blinks, eye-motion artifacts, and segmentation failures.

### Defining photoreceptor related boundaries on spectral-domain optical coherence tomography

**Figure 1:**
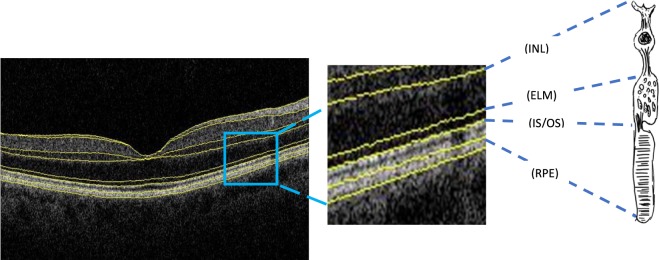
Spectral-domain optical coherence tomography images with schematic showing representative of Inner nuclear layer–Retinal pigment epithelium (INL-RPE); Inner nuclear layer- External limiting membrane (INL-ELM); External limiting membrane-Inner and outer segments (ELM-ISOS); and Inner and outer segments-Retinal pigment epithelium thickness (ISOS-RPE).

The TABS segmentation algorithm was used to segment the photoreceptor layer: inner nuclear layer/outer plexiform layer boundary to retinal pigment epithelium (INL-RPE) and the specific sublayers of the photoreceptor: inner nuclear layer/outer plexiform layer boundary to external limiting membrane (INL-ELM); external limiting membrane-inner segment outer segment (ELM-ISOS); and inner segment outer segment-retinal pigment epithelium (ISOS-RPE). (Fig. 1) The INL-RPE is a proxy measure for the total length of a photoreceptor including the outer plexiform layer (OPL), outer nuclear layer (ONL), external limiting membrane (ELM), inner segment (IS) and outer segment (OS) of the photoreceptors. The INL-ELM is a proxy measure of the synaptic terminals, axons and the nucleus of the photoreceptors that includes OPL and ONL. The ELM-ISOS and ISOS-RPE are proxy measures of the IS and OS of the photoreceptors respectively. The anatomy of the sub-photoreceptor layers correlates with the OCT boundaries observed in the retina, hence the sub-photoreceptor layers have been defined using the specific definitions^4^. Some of the boundaries are controversial and in view of evidence from previous studies, the ISOS junction may actually originate from the inner segment ellipsoids, and is sometimes referred to as IS ellipsoid zone (ISeZ) in more recent research reports^4,5^. The ISeZ zone may be formed by mitochondria within the ellipsoid layer of the outer portion of the inner segments of the photoreceptors^4,5,28,29^ Hence, the ELM-ISOS and ISOS-RPE may also be proxy measures of the inner segment of the photoreceptors to the mitochondria of the inner segment, and from the mitochondria to the apical RPE, respectively.

### Inclusion and exclusion criteria

All participants who underwent SD OCT as part of the UK Biobank were included in the initial analysis. Exclusion criteria included participants who withdrew their consent, had poor SD OCT signal strength, missing thickness values from any Early Treatment Diabetic Retinopathy Study (ETDRS) subfield, image quality score <45, poor centration certainty, or poor segmentation certainty using TABS software^26^. Participants with the following eye conditions were also excluded from the study: refractive error >+6 diopters (D) or <−6D; visual acuity worse 0.1 logMAR; IOPcc of <6 mmHg or >21 mmHg; self-reported glaucoma, ocular disorders, diabetes or neurodegenerative disease.

### Statistical analysis

For this analysis, if both eyes of a patient were eligible for inclusion, one eye was randomly selected using STATA software. Mean, standard deviations (SD) and 95% confidence intervals of the INL-RPE, INL-ELM, ELM-ISOS and ISOS-RPE thickness by ETDRS subfield were calculated for all participants, subsets of demographic and ocular variables. The ETDRS subfields consisted of the “central subfield”, 1 mm diameter from the centre of the fovea; “inner subfield”, 1 to 3 mm from the centre of the fovea and the “outer subfield”, 3 to 6 mm from the centre of the fovea. Both the inner and outer subfields were measured in four sectors (superior, inferior, nasal and temporal).

First, univariate models were constructed examining the association of risk factors (age, sex, race, smoking status, systolic blood pressure (SBP), refraction, IOPcc and corneal hysteresis) with INL-RPE, INL-ELM, ELM-ISOS and ISOS-RPE thicknesses among the sectors (outcomes). Subsequently, multivariate models were constructed additionally adjusting for other confounders. Both unstandardized (*B*) and standardized beta coefficients (β) are shown. The standardized beta coefficient represents the amount of standard deviation change in dependent variable with every 1 SD increase in independent, with the other variables held constant. All *P* values are two-sided and were considered statistically significant when less than 0.05. Data analysis was performed with STATA software version 13.0 (StataCorp LP, College Station, TX, USA).

### Ethical approval

The North West Multi-center Research Ethics Committee approved the study (reference no., 06/MRE08/65), in accordance with the tenets of the Declaration of Helsinki. Detailed information about the study is available at the UK Biobank web site (www.ukbiobank.ac.uk).

## Results

**Figure 2:**
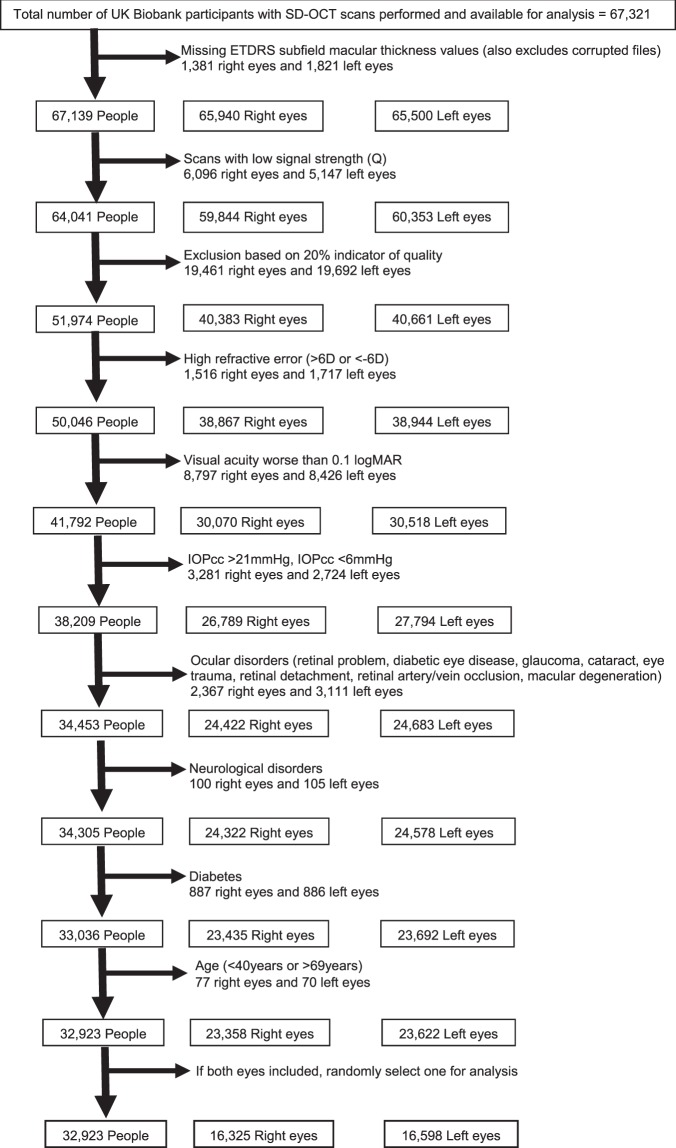
Flowchart showing photoreceptor inclusion and exclusion criteria. D = dioptre; EDTRS = Early Treatment Diabetic Retinopathy Study; IOP = intraocular pressure; logMAR = logarithm of the minimum angle of resolution; OCT = optical coherence tomography; SD = spectral-domain.

Of the 502,656 participants in the whole UK Biobank cohort, 133,668 underwent eye examination. Of these, 67,321 participants had SD OCT macular imaging available for analysis at the time of this report. Of the 67,321, there were 51,974 participants with high-quality images. Of these, 19,051 people with high refractive error (>6D or <−6D), visual acuity worse than 0.1 logMAR, IOPcc of <6 mmHg or >21 mmHg, self-reported eye diseases, neurological disorders and diabetes, and participants <40 years or >69 years old were excluded. Thus, the final sample size for the current analysis was 32,923 (Fig. 2).

Table 1 shows the distribution of demographics and ocular factors among participants included in the analysis (n = 32,923). The mean age of participants was 55.2 years (range 40 to 69 years), 54% were females, majority were White (92%), had higher education level of degree or above (42%), did not smoke (56%), with mean Townsend index of −1.1 (range −6.3 to 8.9) and mean height of 169.2 cm (range 122 to 201 cm). Ocular factors included visual acuity (mean = −0.07 logMAR, range = −0.66 to 0.10 logMAR), refractive error (mean = −0.07D, range = −6 to 6D) and IOPcc (mean = 15.2 mmHg, range 6 to 21 mmHg). The mean systolic blood pressure and diastolic blood pressure was 138.3 mmHg (range 74 to 241 mmHg) and 81.7 mmHg (range 46 to 142 mmHg) respectively.

**Table 1:** Demographics of the participants included in the study.

Characteristic	N	Mean ± SD/(%)
Age at recruitment (years)	32,923	55.2 ± 8.2
Sex		
Male	15063	45.8
Females (%)	17860	54.2
Race (%)		
White	30263	92.3
Chinese	108	0.3
Asian	807	2.5
Black	889	2.7
Mixed/other	737	2.2
Education level (%)		
Less than ‘O’ level	1942	6.7
‘O’ level	7030	24.3
‘A’ level	7740	26.8
Degree and above	12213	42.2
Townsend deprivation index	32,886	−1.1 ± 2.9
Height (cm)	32,821	169.2 ± 9.2
Smoking status (%)		
Never	18351	55.9
Former	11334	34.5
Current	3145	9.6
Eye laterality (%)		
Right eye	16325	49.6
Left eye	16598	50.4
Visual acuity (logMAR)	32,923	−0.07 ± 0.09
Refraction (D)	32,923	−0.07 ± 1.91
IOPcc (mmHg)	32,923	15.2 ± 2.9
Systolic blood pressure (mmHg)	32,730	138.3 ± 19.3
Diastolic blood pressure (mmHg)	32,729	81.7 ± 10.6

IOP = intraocular pressure; logMAR = logarithm of the minimum angle of resolution; SD = standard deviation.

**Figure 3:**
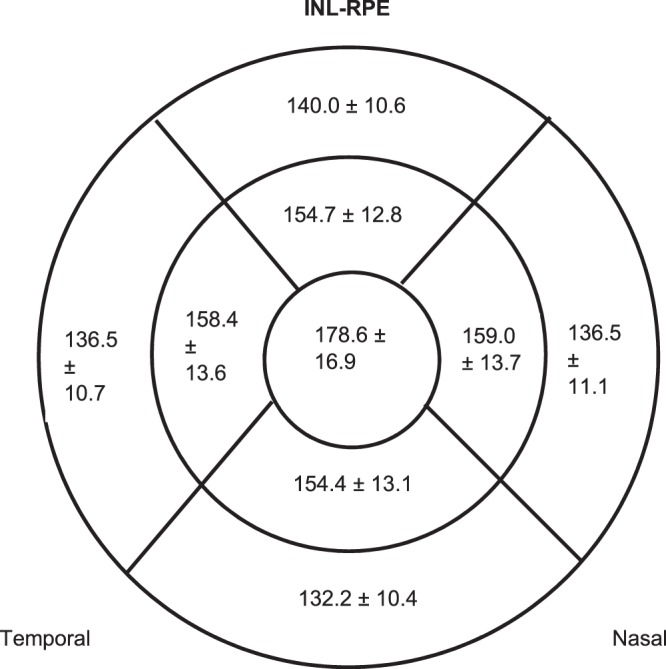
Diagrams showing Inner nuclear layer–Retinal pigment epithelium (INL-RPE) thickness (µm) at the central, inner and outer subfields across the 4 sectors.

The mean and SDs of the INL-RPE thickness in the 9 ETDRS subfields are shown in Fig. 3. The thickness of the specific sublayers (INL-ELM, ELM-ISOS and ISOS-RPE) are shown in Fig. S1. The centre INL-RPE layer is the thickest (178.6 ± 16.9 µm), followed by INL-ELM (107.7 ± 12.5 µm), ISOS-RPE (42.5 ± 7.1 µm) and ELM-ISOS (28.3 ± 2.8 µm). The inner subfields were thicker than the outer subfields for all the photoreceptor layers (INL-RPE, INL-ELM, ELM-ISOS and ISOS-RPE) (P < 0.001), except for the superior and nasal subfields of ISOS-RPE (P < 0.01).

**Figure 4:**
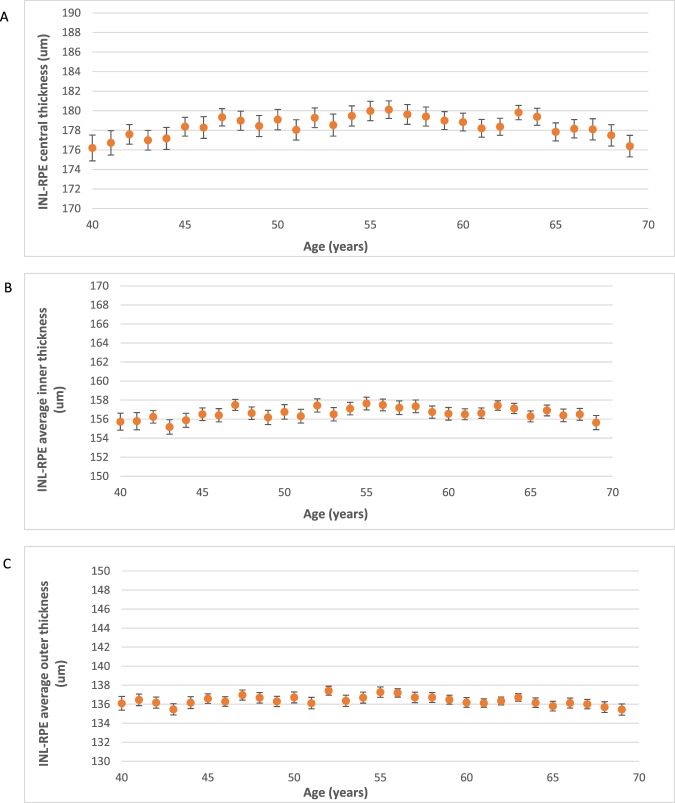
Graphs showing the mean Inner nuclear layer–Retinal pigment epithelium (INL-RPE) thickness (µm) in the (**A**) central, (**B**) average inner, and (**C**) average outer subfields by age. Error bars indicate 95% confidence interval.

**Figure 5:**
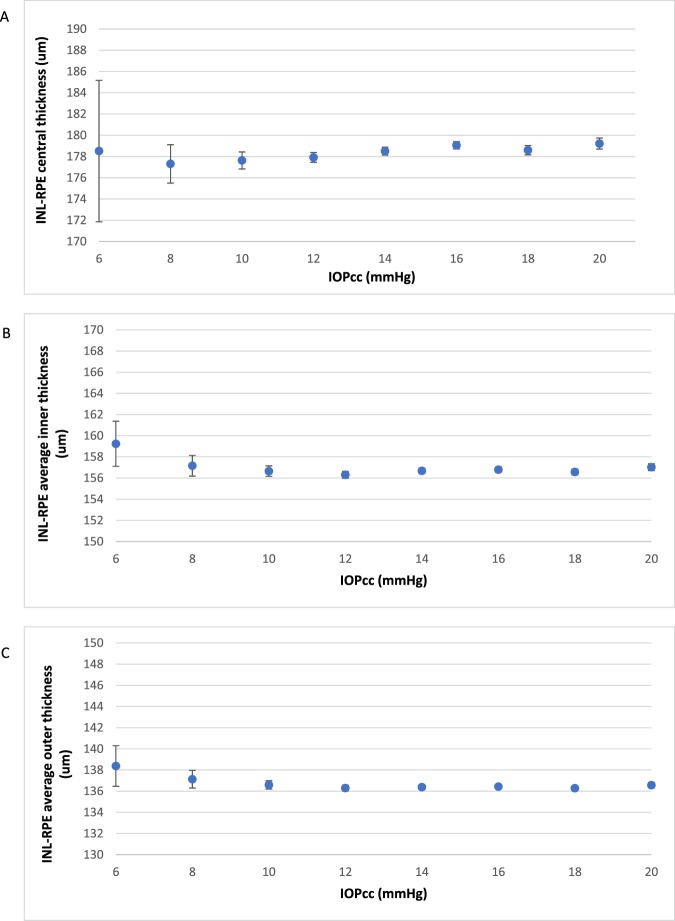
Graphs showing the mean Inner nuclear layer–Retinal pigment epithelium (INL-RPE) thickness (µm) in the (**A**) central, (**B**) average inner, and (**C**) average outer subfields by IOPcc (mmHg). Error bars indicate 95% confidence interval.

**Figure 6:**
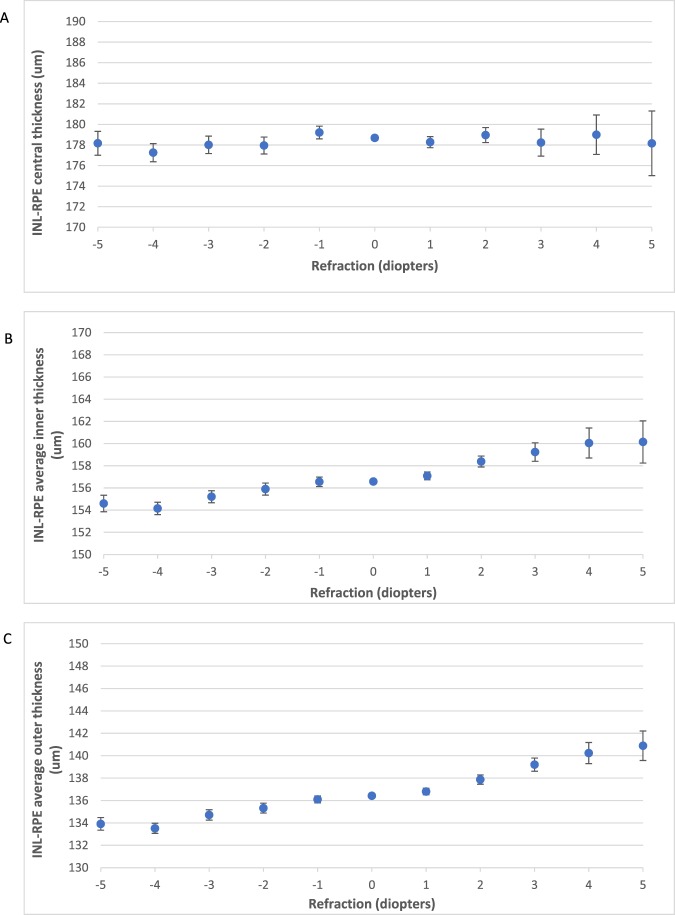
Graphs showing the mean Inner nuclear layer–Retinal pigment epithelium (INL-RPE) thickness (µm) in the (**A**) central, (**B**) average inner, and (**C**) average outer subfields by refraction (**D**). Error bars indicate 95% confidence interval.

Figure 4 shows the change in mean thickness of the INL-RPE layer with age. We found a higher INL-RPE thickness in the central subfield among participants aged 40–54 years compared to participants aged 55–64 years. The inner and outer subfields showed similar trends. In Fig. 5, we found a lower INL-RPE thickness in the central subfield among participants with IOPcc between 6–8 mmHg compared to participants with IOPcc between 8–20 mmHg. Both the average INL-RPE thickness in the inner and outer subfields decreased from 6–12 mmHg and there was no trend from 12–20 mmHg. Figure 6 shows a higher INL-RPE thickness with more positive refractive error (more hyperopia).

Compared to younger participants, the mean overall thickness of INL-ELM is higher, while the thickness of ELM-ISOS is lower in older participants. A higher ISOS-RPE thickness was observed among participants aged 40–54 years compared to participants aged 55–69 years. (Figs. S2–S4). In participants with higher IOPcc, the overall thickness of ELM-ISOS is lower, while the thickness of ISOS-RPE is higher, and there was no association with INL-ELM, compared to participants with lower IOPcc. the mean overall thickness of INL-ELM is higher, while the ELM-ISOS thickness is lower and there was no association with ISOS-RPE (Figs. S8–S10).

Table 2 shows the associations between the risk factors with the thickness of INL-RPE layer after adjusting for age, sex, ethnicity, Townsend deprivation index, height, smoking status, systolic blood pressure, refraction, IOPcc and corneal hysteresis. Older participants had a thinner INL-RPE layer thickness in all subfields (p < 0.001) except the central subfield. Females had thinner INL-RPE compared to males. Compared to those of White ethnicity, those of Black ethnicity had the thinnest INL-RPE in all subfields, followed by Asians and Mixed/Others. Compared to never smokers, previous smokers had thinner INL-RPE in the central and inner subfields, while current smokers had thinner outer and total average subfields. Systolic blood pressure was negatively associated with INL-RPE in all subfields except in the inner subfield. Participants with more positive refraction were associated with thicker INL-RPE. Participants with higher IOPcc were observed to have thicker INL-RPE in all subfields except for the outer subfield. Corneal hysteresis was positively associated with INL-RPE in the outer and total average subfields. Table 3 reports a summary of our findings compared to results of previous studies.

**Table 2:** Multivariate analysis of demographics and risk factors with the thickness of INL-RPE layer.

	Central subfield				Average Inner Subfield				Average Outer Subfield				Total Average			
	β	*B*	95% CI	P	β	*B*	95% CI	P	β	*B*	95% CI	P	β	*B*	95% CI	P
Age	−0.011	−0.023	(−0.048, 0.002)	0.076	−0.020	−0.028	(−0.045, −0.011)	0.001	−0.053	−0.058	(−0.071, −0.045)	<0.001	−0.041	−0.048	(−0.063, −0.034)	<0.001
Sex																
Male		Ref				Ref				Ref				Ref		
Female		−1.944	(−2.479, −1.408)	<0.001		−1.704	(−2.060, −1.347)	<0.001		−1.655	(−1.938, −1.373)	<0.001		−1.684	(−1.988, −1.381)	<0.001
Race																
White		Ref				Ref				Ref				Ref		
Chinese		−3.903	(−7.081, −0.726)	0.016		−0.831	(−2.959, 1.297)	0.444		−0.442	(−2.127, 1.243)	0.607		−0.28	(−2.093, 1.534)	0.762
Asian		−5.868	(−7.070, −4.665)	<0.001		−3.581	(−4.382, −2.780)	<0.001		−2.804	(−3.437, −2.170)	<0.001		−3.01	(−3.691, −2.328)	<0.001
Black		−11.438	(−12.603, −10.273)	<0.001		−7.688	(−8.465, −6.911)	<0.001		−6.122	(−6.738, −5.505)	<0.001		−6.501	(−7.164, −5.837)	<0.001
Mixed/Others		−4.286	(−5.530, −3.042)	<0.001		−2.826	(−3.657, −1.996)	<0.001		−2.426	(−3.084, −1.768)	<0.001		−2.6	(−3.308, −1.892)	<0.001
Smoking status																
Never		Ref				Ref				Ref				Ref		
Previous		0.588	(0.187, 0.988)	0.004		0.279	(0.012, 0.546)	0.041		0.093	(−0.118, 0.304)	0.388		0.157	(−0.070, 0.384)	0.176
Current		−0.08	(−0.729, 0.568)	0.808		−0.298	(−0.729, 0.134)	0.176		−0.558	(−0.899, −0.217)	0.001		−0.54	(−0.907, −0.173)	0.004
SBP (per 10 mmHg)	−0.162	−0.14	(−0.25, −0.04)	0.007	−0.105	−0.06	(−0.13, 0.01)	0.081	−0.208	−0.1	(−0.15, −0.04)	0.001	−0.185	−0.09	(−0.15, −0.03)	0.002
Refraction (per 1D)	0.011	0.121	(0.004, 0.238)	0.042	0.087	0.619	(0.541, 0.697)	<0.001	0.130	0.729	(0.667, 0.791)	<0.001	0.113	0.683	(0.617, 0.750)	<0.001
IOP cornea compensated (mmHg)	0.023	0.135	(0.065, 0.206)	<0.001	0.013	0.049	(0.002, 0.096)	0.039	0.012	0.037	(−0.000, 0.074)	0.052	0.013	0.043	(0.003, 0.083)	0.033
Corneal hysteresis	−0.003	−0.029	(−0.137, 0.080)	0.606	0.008	0.046	(−0.026, 0.118)	0.213	0.016	0.075	(0.018, 0.132)	0.01	0.014	0.072	(0.010, 0.133)	0.022

*Adjusted for age, gender, ethnicity, townsend deprivation index, height, smoking status, systolic blood pressure, refraction, IOP corneal compensated, and corneal hysteresis.

β = standardised beta; *B* = unstandardised beta; P = P value.

**Table 3:** Summary of our findings compared to results of other studies.

Source	Results of our study	Other studies
Effect of age	Thinner INL-RPE, INL-ELM, ELM-ISOS layers with older age	Ooto *et al*. reported thicker central INL-ELM and ISOS-RPE, but thinner ELM-ISOS
	Thicker central INL-ELM layer with older age	Harris *et al*. and Chan *et al*. reported no associations.
	Thinner central ISOS-RPE layer with older age	
Effect of sex	Men had significantly thicker INL-RPE, INL-ELM, ELM-ISOS layers than women	Ooto *et al*. reported men had thicker OPL + ONL layers compared to women.
		Won *et al*. reported men had thicker OPL and thinner ONL compared to women.
Effect of race	Black Britons had thinner INL-RPE, INL-ELM, ELM-ISOS and ISOS-RPE layers compared to Whites	NIL
	Asians and Mixed/Others showed thinner INL-RPE and INL-ELM layers compared to Whites	
Effect of smoking	Current smokers had thinner INL-RPE, ELM-ISOS and ISOS-RPE layers compared to non-smokers	Harris *et al*. reported smokers had thinner photoreceptor thickness in the central and inner subfields compared to non-smokers.
	Previous smokers had thicker INL-RPE and ISO-RPE layers compared to never smokers	
Effect of blood pressure	Higher blood pressure was associated with a thinner INL-RPE and ELM-ISOS	NIL
Effect of refractive error	Higher positive refractive error was associated with thicker INL-RPE and INL-ELM layers.	Ooto *et al*. reported no associations between axial length with OPL + ONL, inner or outer segments of the photoreceptors.
Effect of IOP	Higher IOPcc was associated with thicker INL-RPE, INL-ELM, ELM-ISOS and ISOS-RPE layers.	NIL

Inner nuclear layer/outer plexiform layer boundary to external limiting membrane (INL-ELM); external limiting membrane-inner segment outer segment (ELM-ISOS); and inner segment outer segment-retinal pigment epithelium (ISOS-RPE).

INL-ELM is a proxy measure of the synaptic terminals, axons and the nucleus of the photoreceptors (OPL_ONL). The ELM-ISOS and ISOS-RPE are proxy measures of the inner segment and outer segment of the photoreceptors respectively.

NIL = No other others have been reported and IOP = Intraocular pressure.

Table S1 shows the associations between the risk factors with the thickness of INL-ELM layer after adjusting for similar confounders. Older participants were observed to have thicker INL-ELM in the central subfield, but the outer and total average subfields were thinner. Compared to males, females had thinner INL-ELM in all subfields. Compared to Whites, Blacks had the thinnest INL-ELM in all subfields, followed by Asians and Mixed/Others. No association was observed for smoking status. Systolic blood pressure was negatively associated with INL-ELM thickness in the central subfield. Participants with more positive refraction were associated with thicker INL-ELM in all subfields except for the central subfield. Participants with higher IOPcc were observed to have thicker central INL-ELM.

The multivariate analysis between ocular and systemic variables and the ELM-ISOS thickness are shown in Table S2. Older participants were observed to have thicker ELM-ISOS in all subfields. Females had thinner ELM-ISOS in all subfields compared to males. Compared to Whites, Blacks had the thinnest ELM-ISOS in all subfields, while Mixed/Others had thinner central ELM-ISOS and Chinese had thinner inner and outer subfields. Compared to never smokers, current smokers had thinner ELM-ISOS in all subfields. Systolic blood pressure was negatively associated with ELM-ISOS in all subfields. Participants with more positive refraction were associated with thinner ELM-ISOS in the outer and total average subfields. Participants with higher IOPcc were observed to have thicker central ELM-ISOS. No association was observed for corneal hysteresis.

Table S3 shows the associations between the risk factors with the thickness of ISOS-RPE layer after adjusting for similar confounders. Older participants were observed to have thinner central ISOS-RPE. Females had thicker ISOS-RPE in the central subfield compared to males. Compared to Whites, Blacks had the thinnest ISOS-RPE in all subfields, followed by Mixed/Others. Compared to never smokers, previous smokers had thicker central and inner ISOS-RPE, while current smokers had thinner outer and total average ISOS-RPE. Systolic blood pressure was negatively associated with ISOS-RPE in the outer subfields. Participants with more positive refraction were associated with thinner ISOS-RPE in the central subfields. Participants with higher IOP_cc_ were observed to have thicker ISOS-RPE in all subfields. Corneal hysteresis was positively associated with ISOS-RPE in all subfields.

## Discussion

We report normal photoreceptor thickness metrics for 4 distinct photoreceptor related layers (INL-RPE, INL-ELM, ELM-ISOS and ISOS-RPE) using analysis of the largest known macular SD OCT dataset collected as part of the UK Biobank data resource. Estimating normal retinal photoreceptor layer thickness *in vivo* is important when attempting to distinguish changes due to disease from differences in photoreceptor thickness due to normal variation. In addition, we report how these thickness values vary with ocular and systemic variables across 9 macular sectors. The mean central subfield thickness of the four photoreceptor related retinal layers (INL-RPE, INL-ELM, ELM-ISOS and ISOS-RPE) was the thickest compared to more peripheral macular subfields, as expected in normal anatomy, partially due to the elongation of cone photoreceptors at the fovea. The mean thickness of individual retinal layers in our study were similar to those obtained in other studies of healthy eyes^15–17^. In our study, the mean central INL-ELM (107.7 ± 12.5 µm), ELM-ISOS (28.3 ± 2.8 µm) and ISOS-RPE (42.5 ± 7.1 µm) thickness was similar to a study in 256 Japanese participants (103.9 ± 10.6 µm, 26.9 ± 2.3 µm and 39.8 ± 8.2 µm, respectively)^15^. In addition, the mean sectoral thickness (super, inferior, nasal and temporal) of the 3 retinal layers at the inner and outer subfields were similar^15^. Likewise, a study among 15 participants showed similar results of 42 ± 0.5 µm in the mean central ISOS-RPE thickness^17^. Similar results by Loduca *et al*.^17^ reported the mean central thickness of photoreceptor outer segment was 42.5 ± 5 µm, and was comparable to the results of our study (42.5 ± 7.1 µm).

### Association between age and photoreceptor layer

Our results showed that, in older UK Biobank participants, the INL-RPE, INL-ELM, ELM-ISOS layers were thinner, except for an increased thickness in central INL-ELM, and no significant association for central INL-RPE. These findings were consistent with a study by Ooto *et al*. in 256 healthy Japanese participants aged 20 years and older^15^, which showed a thicker central INL-ELM and thinner ELM-ISOS with older age. Evidence from histologic studies showed outside the foveal centre, photoreceptor thickness decreased significantly with increasing age^30^. Furthermore, rods are affected earlier compared to cones and the decline in photoreceptor density was more marked for rods than for cones (0.37% vs. 0.18%/year, respectively)^31^. With older age, our study showed that the thickness of ISOS-RPE was thinner in the central and no association was observed for the other subfields. In contrast, the study by Ooto *et al*. showed the outer segment of photoreceptors thicken with age^15^. Another study by Harris *et al*. in a Danish population of 150 individuals reported no difference between age and photoreceptor thickness (defined as thickness from ELM-RPE)^19^. The difference in findings may be due to variation in population characteristics such as ethnicity and age group, sample size and the type of confounders adjusted in the multivariate models. In contrast, Chan *et al*. did not report an association between age and outer retinal complex (outer nuclear layer and the inner segment of the photoreceptor layer), but the absence of an association could be due to the small sample size (n = 37)^11^.

### Association between sex and photoreceptor layer

Our finding that men had significantly thicker INL-RPE, INL-ELM, ELM-ISOS than women is consistent with previous study by Ooto *et al*.^15^ Ooto *et al*. reported the outer plexiform layer (OPL) + outer nuclear layer (ONL) was significantly thicker in men than women, however, no differences were observed for inner and outer segments of the photoreceptor layer. In another study by Won *et al*. in 50 Korean participants aged 20–80 years, men had thicker OPL and thinner ONL compared to women. Compared to our study, both studies by Ooto *et al*. and Won *et al*. had significantly fewer participants, the participants were recruited from Asia, consisted of a different age range and had different exclusion criteria^15,16^. In addition, Won *et al*. used a Spectralis, Heidelberg OCT instrument compared to the Topcon OCT instrument used by Ooto *et al*. and our study. It is possible that there are differences in segmentation algorithms in defining retinal layers when different OCT instruments are used to collect data^32^. Angle of incidence of the scanning beam on the retina, leading to differential results in the imaging of the Henle fiber layer, could also be a factor^33,34^. Another explanation for the sex difference could be due to longer axial length observed in men compared to women^35^. However, we did not measure axial length in our study and was unable to evaluate further.

### Association between race and photoreceptor layer

Previous studies that examined race-related differences were only performed for total macular thickness^36,37^. Compared to Whites, Africans and African Americans had reduced macular thickness. Similarly, our study showed that Black Britons had thinner INL-RPE, INL-ELM, ELM-ISOS and ISOS-RPE compared to Whites. In addition, Asians and Mixed/Others showed thinner INL-RPE and INL-ELM compared to Whites. Though the precise reason for the association between ethnicity and photoreceptor length is unclear, one previously cited reason is that the higher concentrations of melanin in the RPE among black participants may alter optical reflectivity at the photoreceptor-RPE interface leading to an underassessment of retinal thickness in individuals with darker skin colour^37,38^.

### Association between smoking and photoreceptor layer

Our study showed that compared to participants who did not smoke, the total average of the INL-RPE, ELM-ISOS and ISOS-RPE layers were thinner in current smokers, but there was no significant difference in the INL-ELM (although a similar trend was evident). This finding supports the observation from histology that cigarette smoke exposure leads to disorganized photoreceptor anatomy and thinner photoreceptor layers^39^. Cigarette smoke is known to contain toxic compounds and causes oxidative damage, vascular and inflammatory changes to the RPE^40^. Therefore, alterations in the metabolic support of the RPE cause apoptosis of the photoreceptors. In agreement with our findings, smokers were observed to have significantly thinner photoreceptor thickness in the central and inner subfields in a Danish population^19^. In contrast, previous smokers showed increased thickness in the INL-RPE and ISO-RPE layer compared to never smokers.

### Association between blood pressure and photoreceptor layer

Our study showed that higher blood pressure was associated with a thinner INL-RPE and ELM-ISOS. Although oxygenation of the avascular outer retina is mainly from the choriocapillaris^41^, the deep capillary plexus (DCP) contributes 10–15% of the oxygen supply to photoreceptor cells^42^. Furthermore, systemic diseases such as hypertension, diabetes, hyperlipidemia may cause changes in blood pressure, resulting in ischaemia of the retinal capillary layers, particularly the DCP^42^. Therefore, a possible explanation of our finding of thinner photoreceptor related retinal layers with increasing blood pressure could be through higher systolic blood pressure leading to intermittent reduction in blood flow at the DCP, leading to subtle changes in photoreceptor layer thickness on OCT imaging. An alternative explanation is that severe hypertension leads to choroidal permeability changes, which increases choroidal interstitial fluid and extends into the subretinal space causing subretinal fluid accumulation, and subsequent photoreceptor defects^43,44^.

### Association between refractive error and photoreceptor layer

Our study showed that more positive refractive error was associated with thicker photoreceptor thickness (INL-RPE and INL-ELM). There were no studies which examined the association between refractive error and photoreceptor thickness, but Ooto *et al*. examined the effect of axial length with photoreceptor thickness^15^. Axial length (AL) has the strongest correlation with refractive status, with more negative refractive error having longer eyes and more positive refractive error have shorter eyes^45^. Previous studies showed longer AL was associated with reduced macular thickness^46,47^. Decreased photoreceptor thickness in participants with longer AL is compatible with histologic findings that showed an elongation of the eyeball leads to mechanical stretching and thinning of the sclera and retina^48^. A possible explanation for our findings is a more positive error may be more associated with shorter AL, hence a thicker photoreceptor thickness. In contrast to our findings, Ooto *et al*. did not report a significant association between axial length with OPL + ONL, inner or outer segments of the photoreceptors^15^. Ooto *et al*. and the authors recruited a smaller study population of 256 participants and an age range of 20–77 years, while our sample size was 32,923 and an age range of 40–69 years^15^. Hence, these factors may explain the difference in findings between our study and Ooto *et al*. Our results showed similar findings where the average inner, average outer and total average of the photoreceptor thickness decreased with longer AL. Although in our study, increased myopic refractive error was associated with thicker photoreceptor thickness (INL-RPE) at the central subfield, the effect estimates were much lower in the central subfield compared to the average inner, average outer and total average.

### Association between IOP and photoreceptor layer

In this study of participants with IOP_cc_ in the 10–21 mmHg range, we showed that higher IOP_cc_ is associated with thicker INL-RPE and ISOS-RPE. In addition, higher IOP_cc_ results in thicker INL-ELM and ELM-ISOS. A possible explanation could be offered by our previous finding that higher IOP is associated with a thinner RPE-BM complex in the UK Biobank^26^. The RPE is a monolayer of pigmented cells located between the photoreceptor layer and choriocapillaris. As the RPE maintains the health and function of the photoreceptors through daily phagocytosis of photoreceptor outer segments^49^, a thinner RPE layer may indicate poorer RPE cell function leading to impaired phagocytosis of photoreceptor outer segments and accumulation of the disc protein. This may explain the positive association between IOP_cc_ and ISOS-RPE in our results. Our study showed that higher cornea hysteresis is associated with a thicker ISOS-RPE at all subfields and thicker average outer subfield and total average of the INL-RPE. This finding may be explained by the relationship between the ability of the cornea to absorb pressure and its impact on the retinal thickness. An eye with high hysteresis may be more flexible and is able to accommodate the increase in pressure, reducing the impact on the photoreceptor thickness. Thus, reflecting higher corneal hysteresis and thicker photoreceptor (INL-RPE) in our results. Further studies are needed to determine the association between corneal hysteresis and photoreceptor thickness.

Our study’s strengths include a large sample size of 32,923 participants, which is 125 times larger than previous studies reporting OCT imaging derived photoreceptor layer thickness^15,16^. This allows weaker associations to be discovered. Other strengths include its standardized methodology and the inclusion of multi-ethnic participants. It’s important to note that the response rate in UK Biobank was 5.5%, and the participants were volunteers. Hence, it is likely that the study participants are likely to be healthier compared to the general UK population. Nonetheless, a broad range of exposures and characteristics would have been captured in the UK Biobank and the findings would still be applicable to other populations with a different distribution of exposures. The self-reported nature of glaucoma, ocular disorders, diabetes or neurodegenerative disease could result in misclassification bias. There is a possibility that people with the disease may be undiagnosed and still be included in the study. Hence, the changes in photoreceptor layer thickness may be driven by specific eye diseases. However, we believe that the effect is minimized as we have also excluded participants with VA worse 0.1 logMAR or Snellen VA worse than 6/7.5. The smoking status was obtained through self-reported questionnaire, but there is less likely to be differential reporting between exposed and non-exposed groups as the questionnaires were administered before the OCT measures. Due to the cross-sectional nature of our study, the causal relationships between the risk factors and photoreceptor thickness cannot be established. Further longitudinal evaluation is required to determine the associations between the risk factors and photoreceptor thickness. Findings related to the photoreceptor layer thickness and the specific sublayers of the photoreceptor rely on the interpretation of the SD OCT imaging. Definition of the ISOS junction may differ across studies^4,5,28,29^, hence, the thickness of the ELM-ISOS and ISOS-RPE defined in the present study and the true thickness of the ELM-ISOS and ISOS-RPE might differ. Although we did not account for Bonferroni correction in this analysis, most of the significant associations had p values < 0.001, which showed strong associations between specific ocular/systemic factors with photoreceptor thickness. However, caution should be applied when interpreting the results of the study for relationships with borderline associations.

In conclusion, our study provides data for photoreceptor thickness measures and examines the associations of various demographic and risk factors with photoreceptor thickness in a large, multi-ethnic sample (UK Biobank). In addition to confirming known associations between age and sex with photoreceptor layer thickness, we report novel findings of the associations of ethnicity, smoking, systolic blood pressure, refraction, IOPcc and corneal hysteresis with photoreceptor thickness measures. Although future prospective studies are needed, our findings may suggest a predisposition of the effect of these risk factors with photoreceptor thickness. Therefore, clinicians may consider the impact of demographics, systemic and ocular factors when evaluating SD OCT derived measures of photoreceptor thickness.

## Supplementary information

Supplementary info
