# Supplementary material for: Associations with photoreceptor thickness measures in the UK Biobank
Source: Sci Rep. 2019 Dec 19;9:19440. doi: 10.1038/s41598-019-55484-1 (PMC6923366; doi:10.1038/s41598-019-55484-1)
Supplement: Supplementary file 1 — Supplementary info [file 41598_2019_55484_MOESM1_ESM.pdf]

Associations with photoreceptor thickness measures in the UK Biobank

Sharon Y.L. Chua PhD[1]; Baljean Dhillon FRCS(Ed) FRCOphth[2,3]; Tariq Aslam FRCSEd.(Ophth), PhD[4,5]; Konstantinos Balaskas FRCOphth[1,6]; Qi Yang PhD[7]; Pearse A. Keane MD[1]; Adnan Tufail FRCOphth[1]; Charles Reisman MSc[7]; Paul J. Foster PhD[1+]; Praveen J. Patel FRCOphth[1+]* on behalf of the UK Biobank Eye and Vision Consortium,

[+]These authors take joint credit for this manuscript

Author affiliations:

[1] NIHR Biomedical Research Centre, Moorfields Eye Hospital NHS Foundation Trust and UCL Institute of Ophthalmology, London, United Kingdom

[2] Centre for Clinical Brain Sciences, School of Clinical Sciences, University of Edinburgh, Edinburgh, UK

[3] NHS Lothian Princess Alexandra Eye Pavilion, Edinburgh, UK

[4] Faculty of Biology, Medicine and Health, School of Pharmacy and Optometry, The University of Manchester, Manchester, UK

[5] Manchester Royal Eye Hospital, NHS Central Manchester University Hospitals, Manchester, United Kingdom

[6] School of Biological Sciences, University of Manchester, Manchester, UK

[7] Topcon Advanced Biomedical Imaging Laboratory, Oakland, New Jersey, United States of America

Corresponding author: Praveen J. Patel

Address: NIHR Biomedical Research Centre, Moorfields Eye Hospital NHS Foundation Trust, 162 City Road, London, EC1V 2PD

Email: Praveen.Patel1@nhs.net

Telephone:

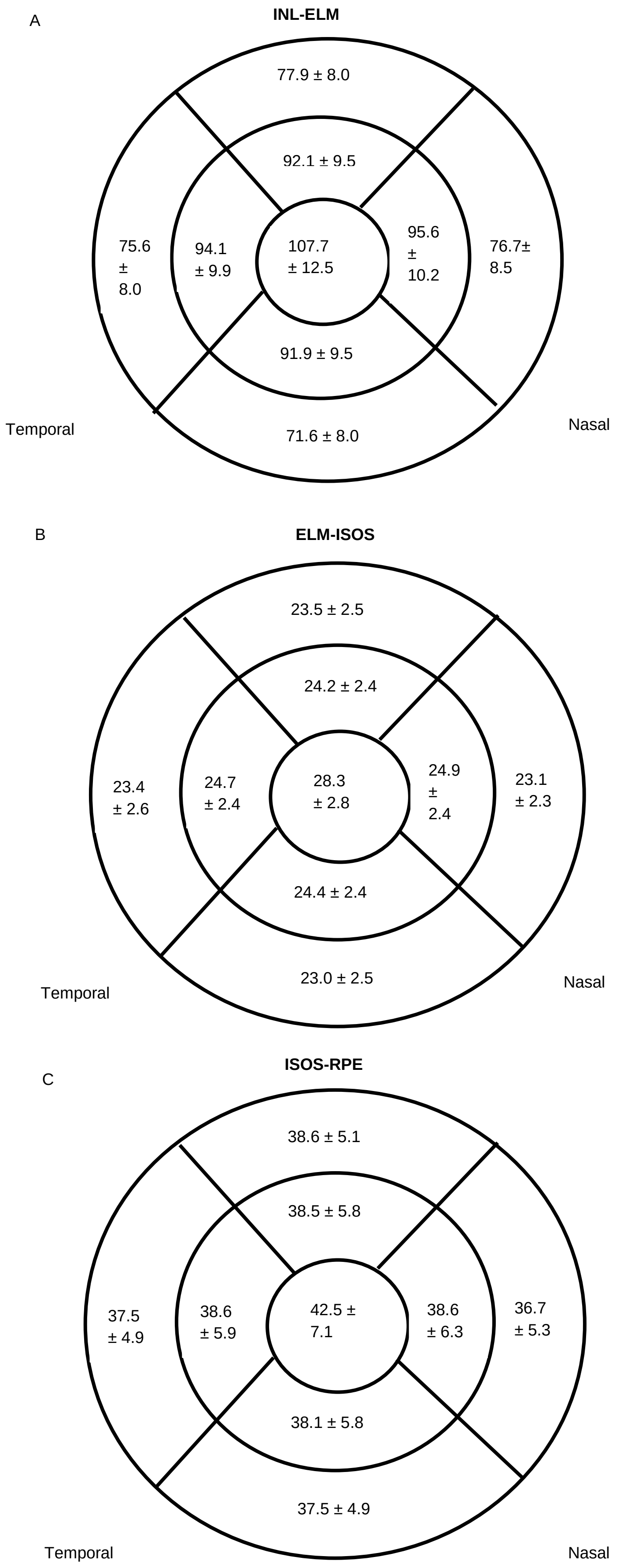

Figure S1. Diagrams showing Inner nuclear layer- External limiting membrane (INL-ELM) thickness (µm) (A), External limiting membrane-Inner and outer segments (ELM-ISOS) thickness (µm) (B) and Inner and outer segments-Retinal pigment epithelium thickness (ISOS-RPE) thickness (µm) (C) at the central, inner and outer subfields across the 4 sectors

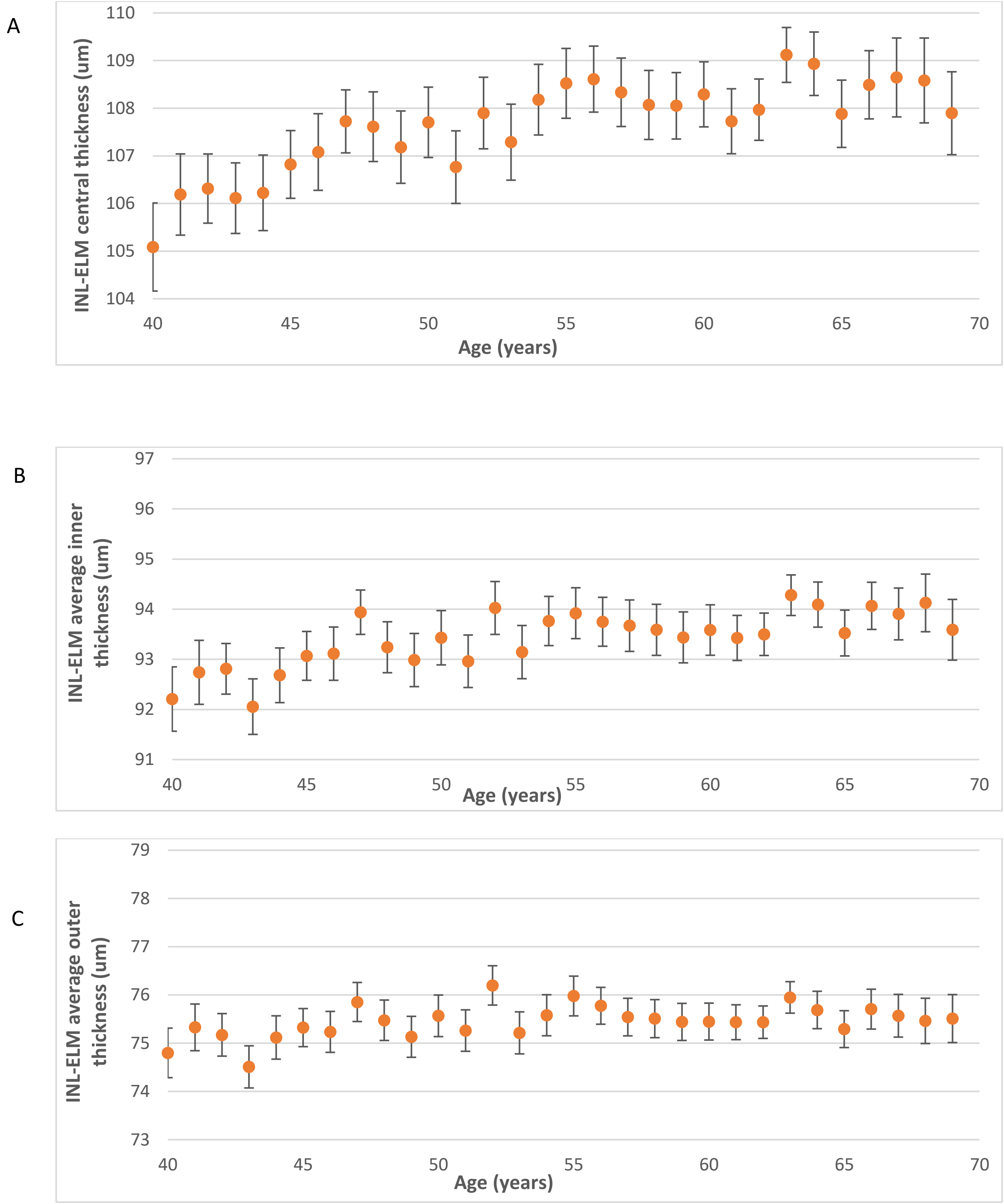

Figure S2. Graphs showing the mean Inner nuclear layer- External limiting membrane (INL-ELM) thickness(μm) in the (A) central, (B) average inner, and (C) average outer subfields by age. Error bars indicate 95% confidence interval.

A

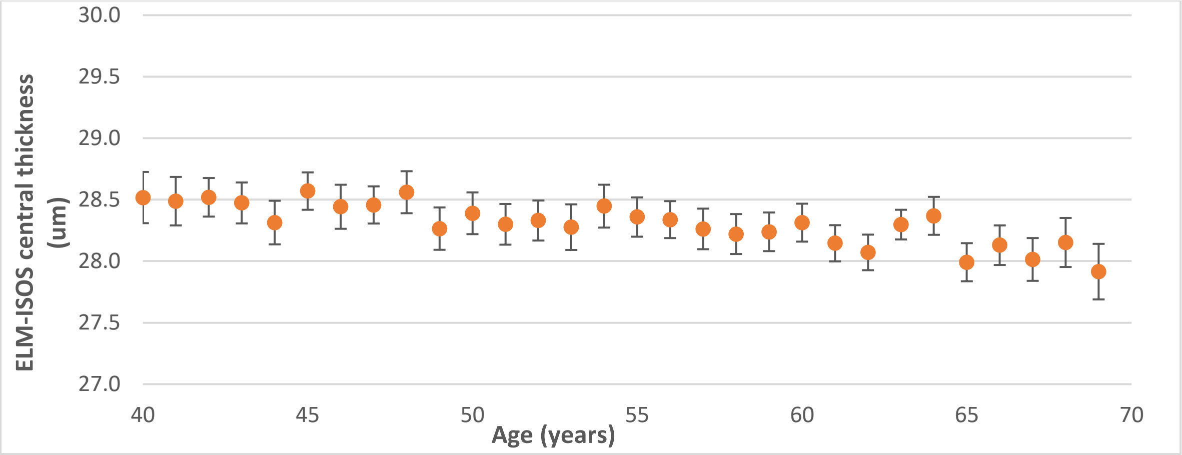

B

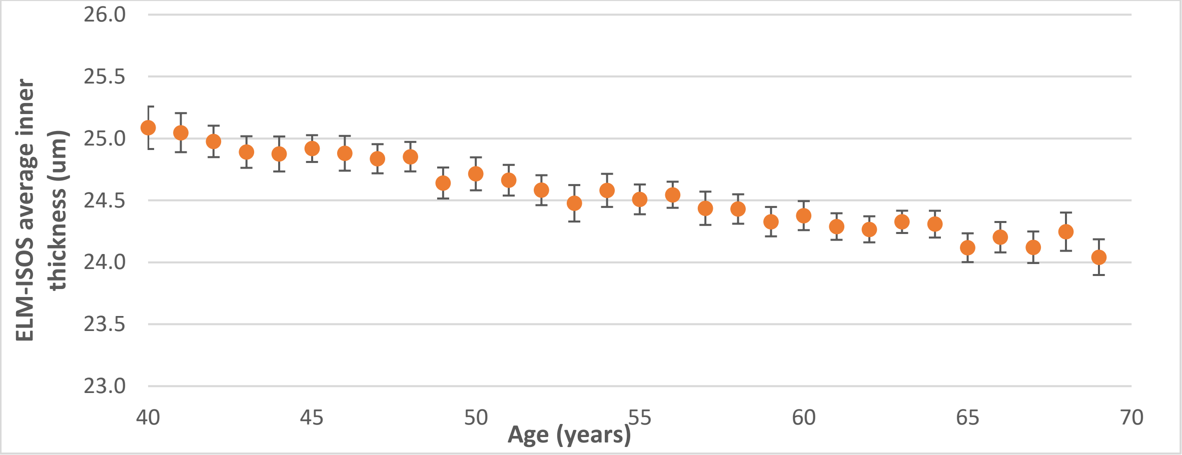

C

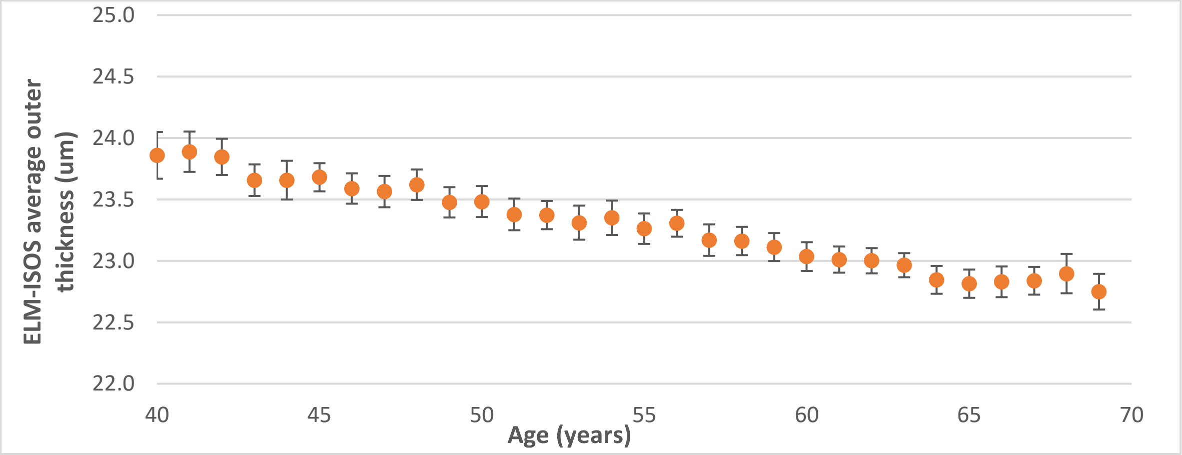

Figure S3. Graphs showing the mean External limiting membrane-Inner and outer segments (ELM-ISOS) thickness (μm) in the (A) central, (B) average inner, and (C) average outer subfields by age. Error bars indicate 95% confidence interval.

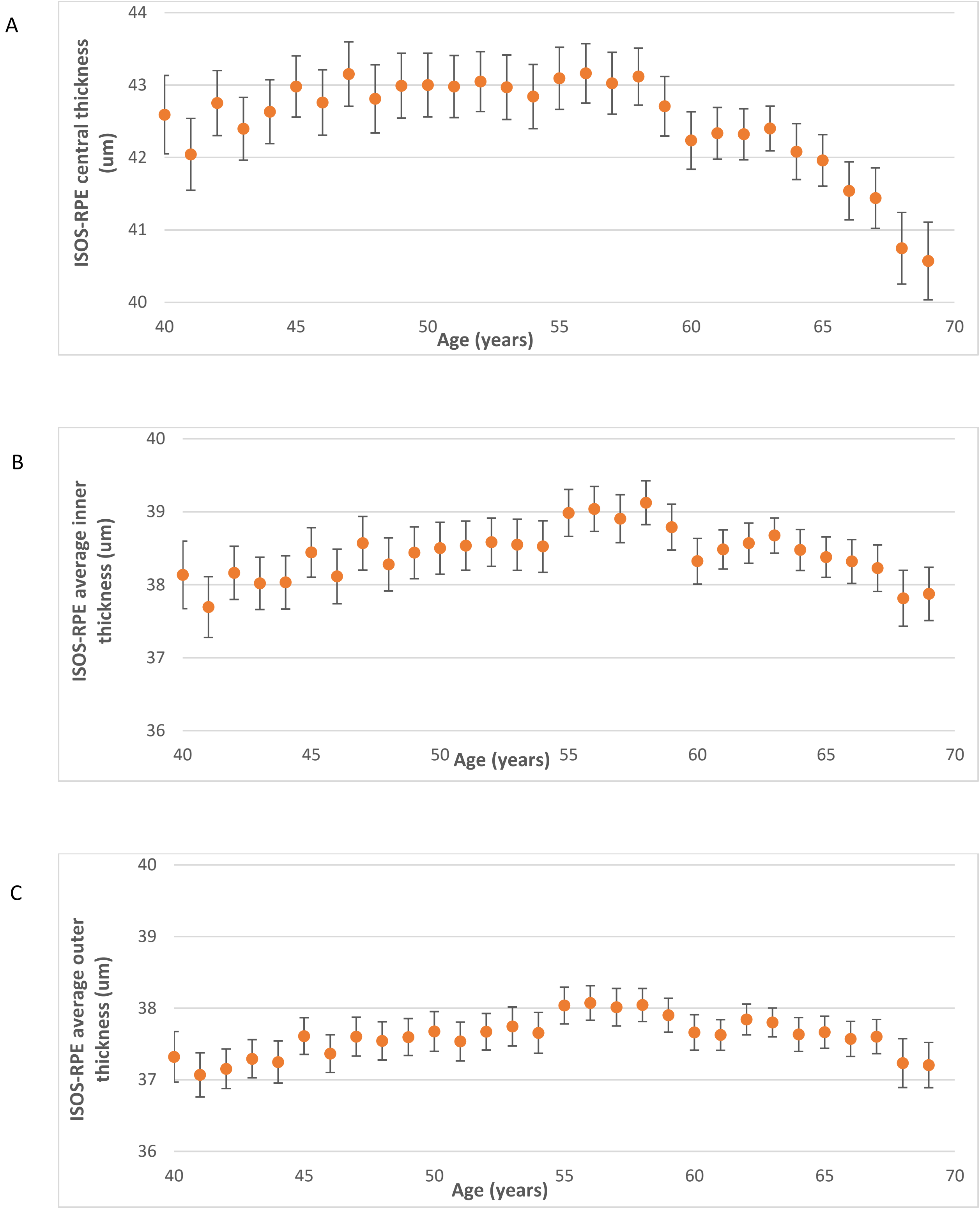

Figure S4. Graphs showing the mean Inner and outer segments-Retinal pigment epithelium thickness (ISOS-RPE) thickness (µm) in the (A) central, (B) average inner, and (C) average outer subfields by age. Error bars indicate 95% confidence interval.

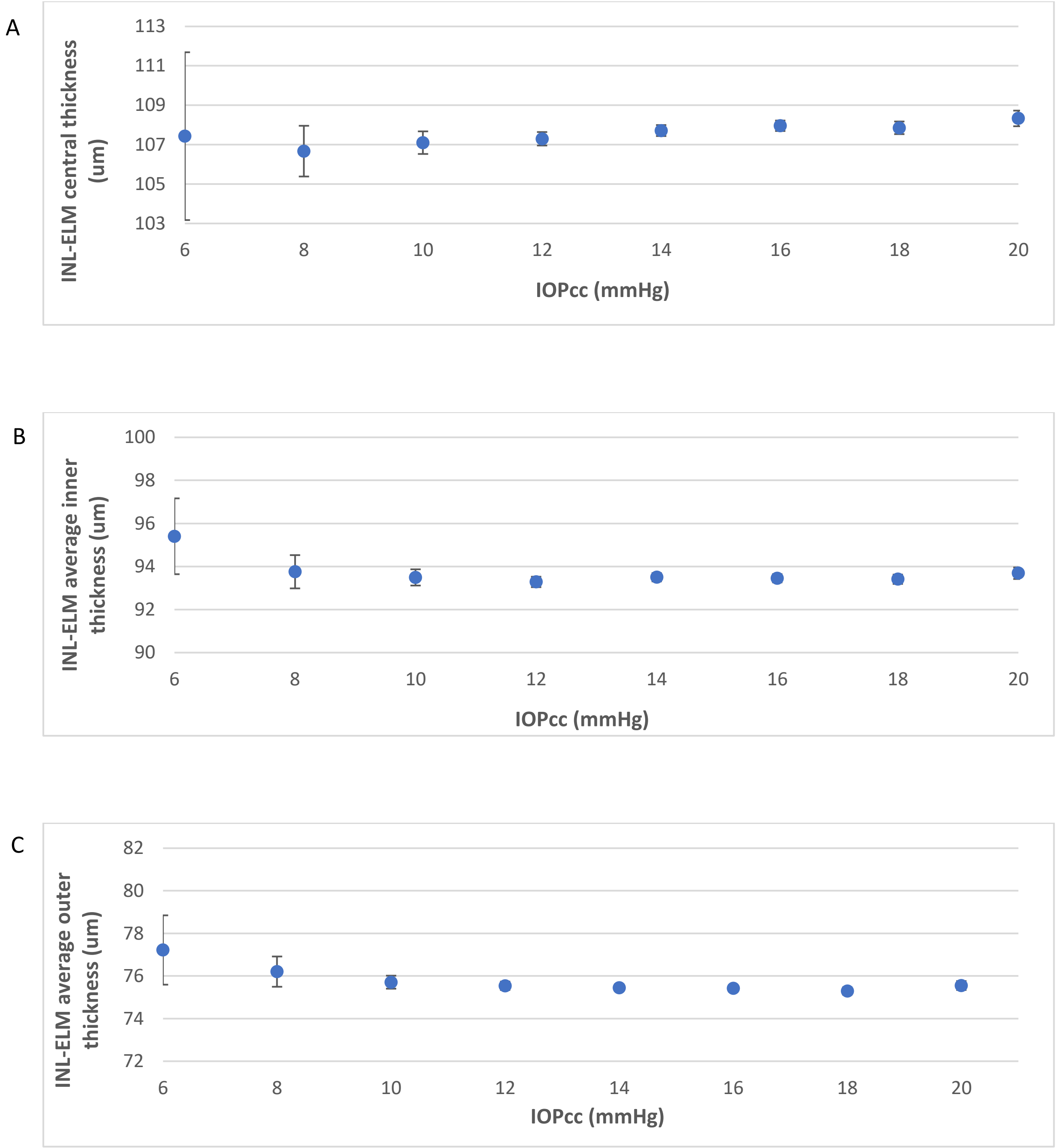

Figure S5. Graphs showing the mean Inner nuclear layer- External limiting membrane (INL-ELM) thickness (µm) in the (A) central, (B) average inner, and (C) average outer subfields by IOP_{cc} (mmHg). Error bars indicate 95% confidence interval.

A

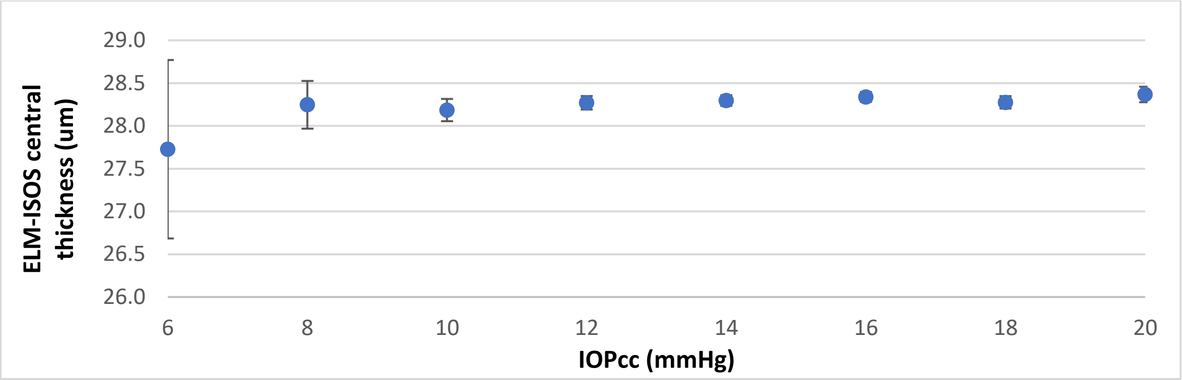

B

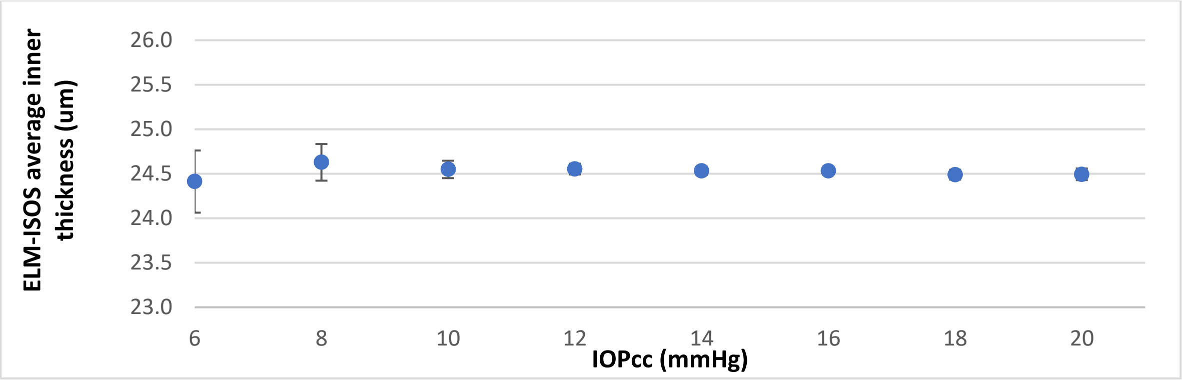

C

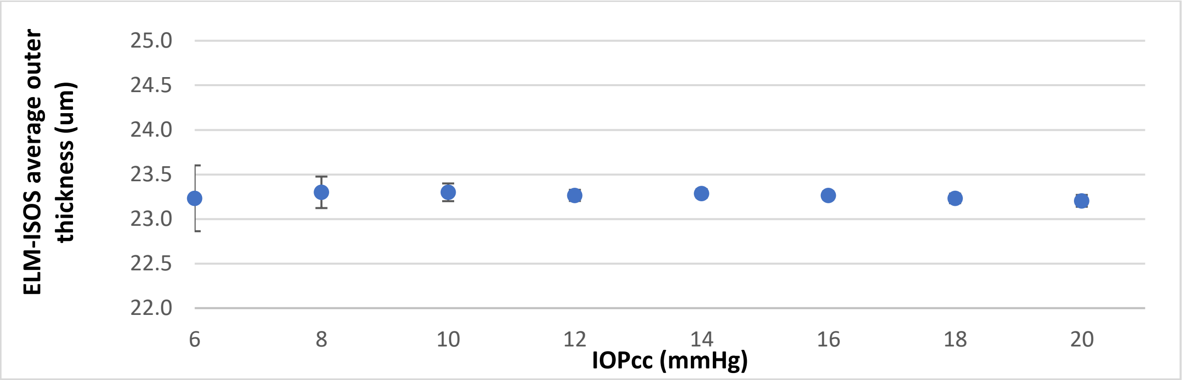

Figure S6. Graphs showing the mean External limiting membrane-Inner and outer segments (ELM-ISOS) thickness (µm) in the (A) central, (B) average inner, and (C) average outer subfields by IOP_{cc} (mmHg). Error bars indicate 95% confidence interval.

A

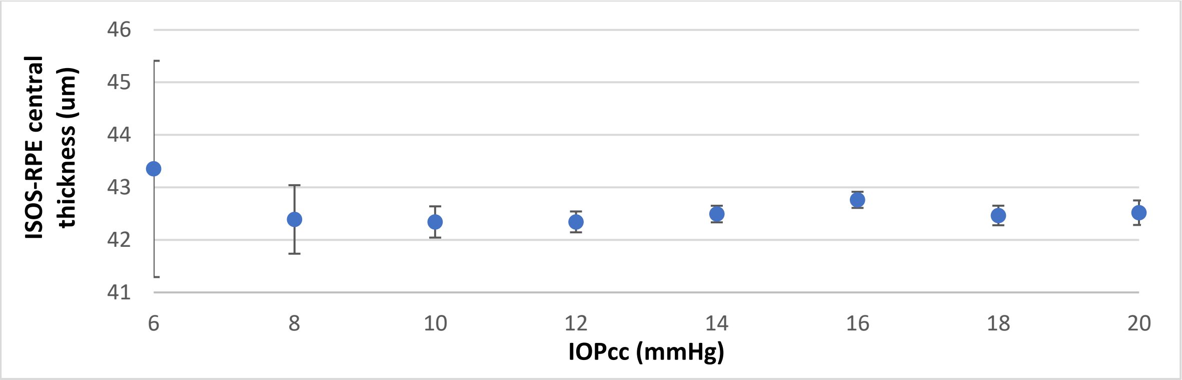

B

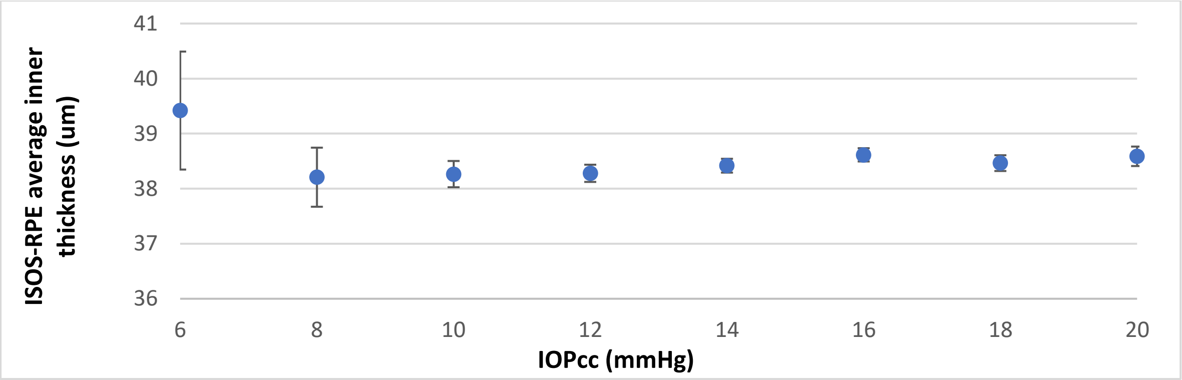

C

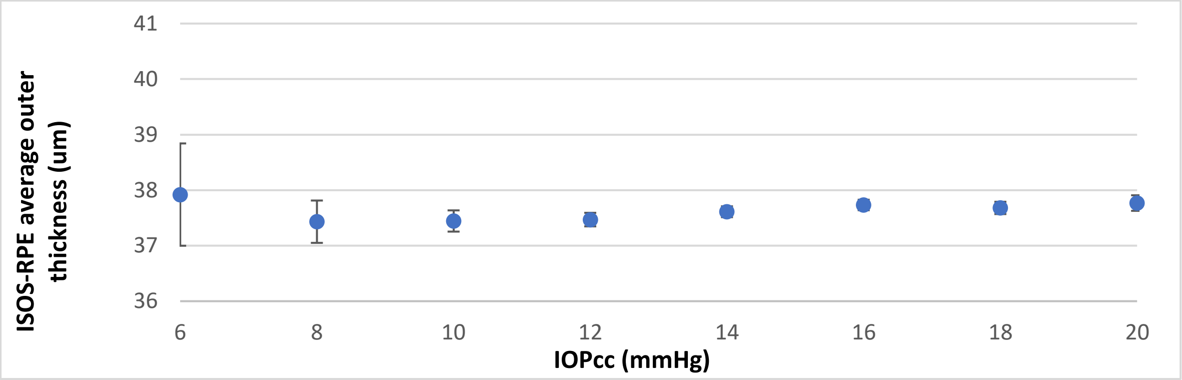

Figure S7. Graphs showing the mean Inner and outer segments-Retinal pigment epithelium thickness (ISOS-RPE) thickness (µm) in the (A) central, (B) average inner, and (C) average outer subfields by IOP_{cc} (mmHg). Error bars indicate 95% confidence interval

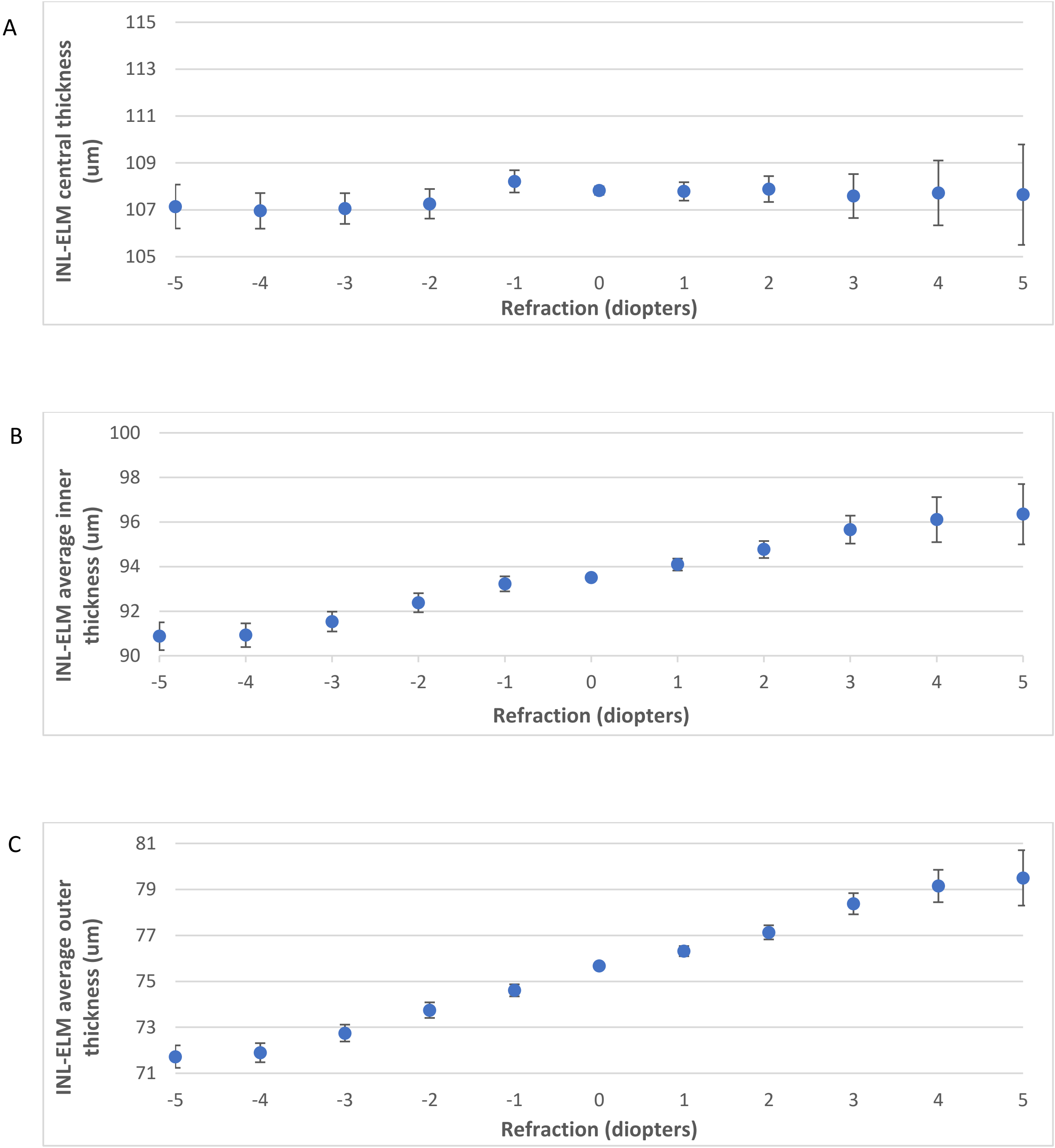

Figure S8. Graphs showing the mean Inner nuclear layer- External limiting membrane (INL-ELM) thickness (µm) in the (A) central, (B) average inner, and (C) average outer subfields by refraction (D). Error bars indicate 95% confidence interval

A

B

C

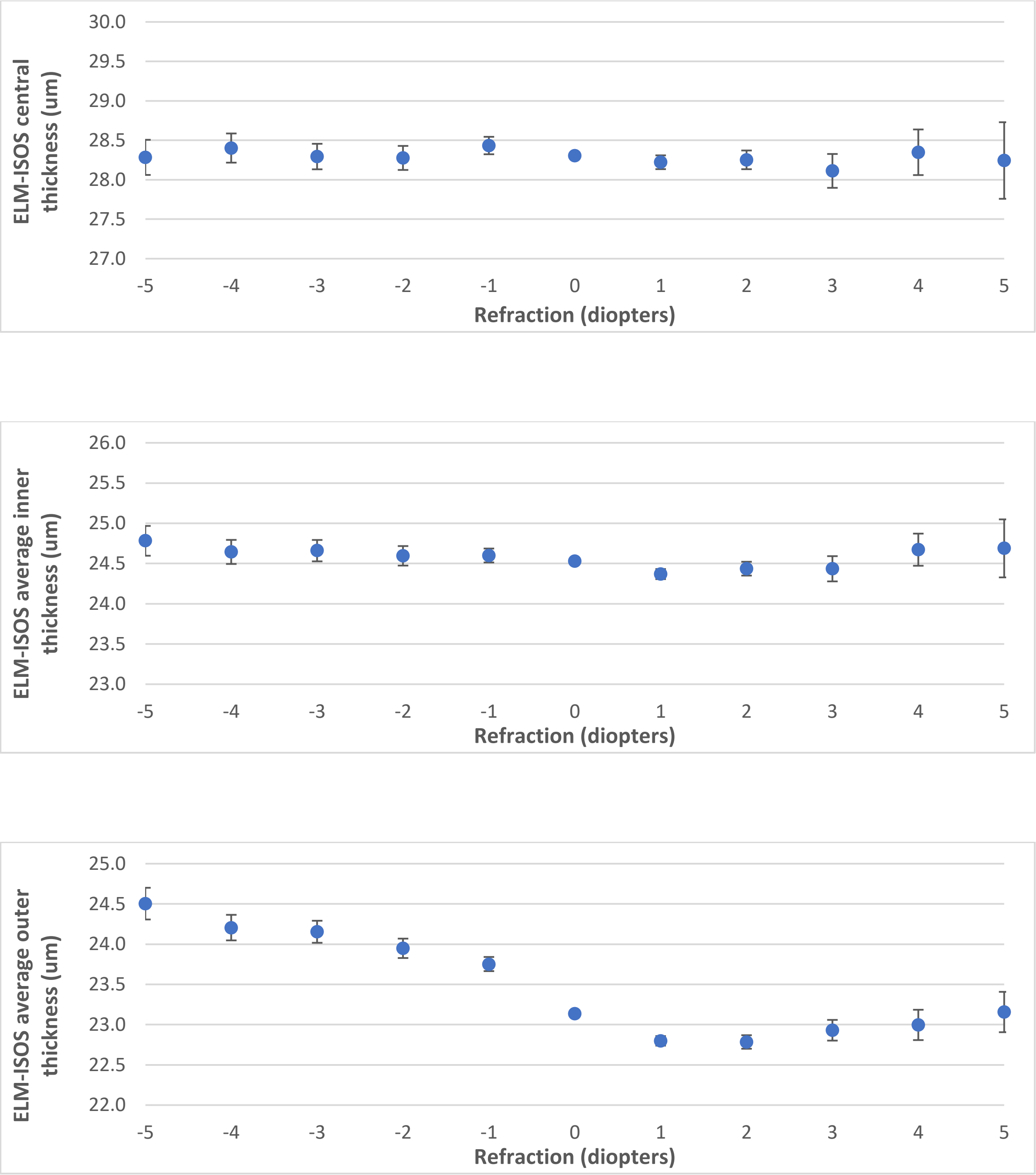

Figure S9. Graphs showing the mean External limiting membrane-Inner and outer segments (ELM-ISOS) thickness (µm) in the (A) central, (B) average inner, and (C) average outer subfields by refraction (D). Error bars indicate 95% confidence interval

A

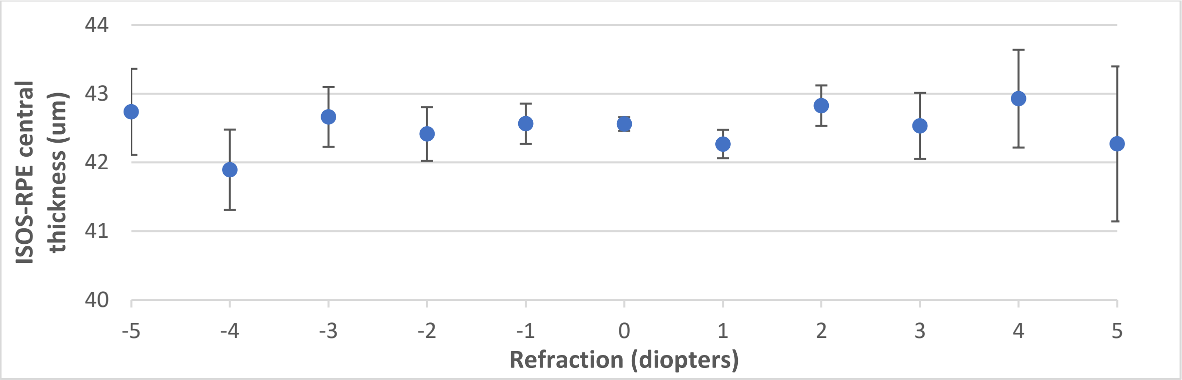

B

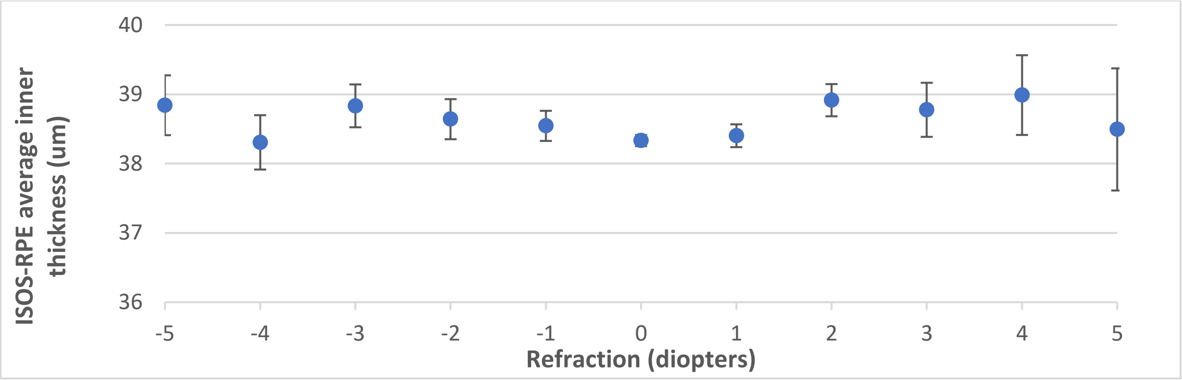

C

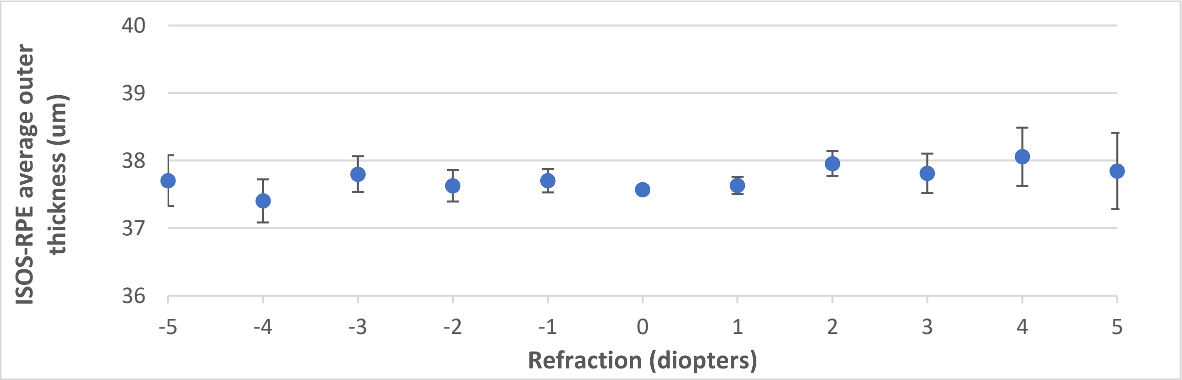

Figure S10. Graphs showing the mean Inner and outer segments-Retinal pigment epithelium thickness (ISOS-RPE) thickness (µm) in the (A) central, (B) average inner, and (C) average outer subfields by refraction (D). Error bars indicate 95% confidence interval.

Table S1. Multivariate analysis of demographics and risk factors with the thickness of INL-ELM layer

	Central subfield				Average Inner Subfield				Average Outer Subfield				Total Average			
	β	*B*	95% CI	P	β	*B*	95% CI	P	β	*B*	95% CI	P	β	*B*	95% CI	P
Age	0.03	0.05	(0.03, 0.07)	<0.001	0.003	0.003	(-0.009, 0.02)	0.60	-0.037	-0.03	(-0.04, -0.02)	<0.001	-0.024	-0.02	(-0.03, -0.01)	<0.001
Sex																
Male		Ref				Ref				Ref				Ref		
Female		-2.18	(-2.58, -1.79)	<0.001		-1.42	(-1.70, -1.15)	<0.001		-1.39	(-1.60, -1.17)	<0.001		-1.43	(-1.66, -1.21)	<0.001
Race																
White		Ref				Ref				Ref				Ref		
Chinese		-4.67	(-7.02, -2.33)	<0.001		-2.44	(-4.05, -0.82)	0.003		-1.23	(-2.52, 0.06)	0.06		-1.30	(-2.64, 0.04)	0.06
Asian		-5.60	(-6.49, -4.71)	<0.001		-3.78	(-4.39, -3.17)	<0.001		-2.60	(-3.09, -2.11)	<0.001		-2.97	(-3.48, -2.47)	<0.001
Black		-7.38	(-8.24, -6.52)	<0.001		-4.26	(-4.85, -3.66)	<0.001		-3.58	(-4.05, -3.11)	<0.001		-3.67	(-4.15, -3.18)	<0.001
Mixed/Others		-2.95	(-3.87, -2.03)	<0.001		-1.75	(-2.39, -1.12)	<0.001		-1.44	(-1.94, -0.93)	<0.001		-1.55	(-2.07, -1.02)	<0.001
Smoking status																
Never		Ref				Ref				Ref				Ref		
Previous		0.30	(0.002, 0.59)	0.05		0.16	(-0.05, 0.36)	0.14		0.05	(-0.12, 0.21)	0.60		0.09	(-0.08, 0.26)	0.28
Current		0.12	(-0.36, 0.60)	0.63		0.13	(-0.20, 0.46)	0.44		-0.05	(-0.32, 0.21)	0.70		-0.04	(-0.31, 0.23)	0.78
SBP (per 10mmHg)	-0.12	-0.08	(-0.16, -0.004)	0.04	-0.07	-0.03	(-0.08, 0.02)	0.25	-0.11	-0.04	(-0.08, 0.002)	0.06	-0.10	-0.04	(-0.08, 0.01)	0.09
Refraction (per 1D)	0.005	0.04	(-0.05, 0.13)	0.38	0.11	0.60	(0.54, 0.66)	<0.001	0.20	0.89	(0.84, 0.94)	<0.001	0.18	0.81	(0.76, 0.86)	<0.001
IOP_{cc} (mmHg)	0.01	0.06	(0.003, 0.11)	0.04	0.003	0.009	(-0.03, 0.05)	0.61	-0.001	-0.003	(-0.03, 0.03)	0.82	0.0001	0.0003	(-0.03, 0.03)	0.99
Corneal hysteresis	-0.01	-0.08	(-0.16, -0.002)	0.05	0.0004	0.0002	(-0.06, 0.06)	0.99	0.01	0.03	(-0.02, 0.07)	0.22	0.006	0.02	(-0.02, 0.07)	0.34

Adjusted for age, gender, ethnicity, Townsend deprivation index, height, smoking status, systolic blood pressure, refraction, IOP_{cc}, and corneal hysteresis
β = standardised beta; *B*= unstandardised beta; P= P value; SBP= systolic blood pressure; IOP_{cc}= Corneal-compensated intraocular pressure

Table S2. Multivariate analysis of demographics and risk factors with the thickness of ELM-ISOS layer

	Central subfield				Average Inner Subfield				Average Outer Subfield				Total Average			
	β	*B*	95% CI	P	β	*B*	95% CI	P	β	*B*	95% CI	P	β	*B*	95% CI	P
Age	-0.06	-0.02	(-0.02, -0.02)	<0.001	-0.13	-0.03	(-0.04, -0.03)	<0.001	-0.11	-0.03	(-0.03, -0.03)	<0.001	-0.13	-0.03	(-0.03, -0.03)	<0.001
Sex																
Male		Ref				Ref				Ref				Ref		
Female		-0.30	(-0.39, -0.22)	<0.001		-0.30	(-0.37, -0.23)	<0.001		-0.23	(-0.29, -0.16)	<0.001		-0.25	(-0.31, -0.18)	<0.001
Race																
White		Ref				Ref				Ref				Ref		
Chinese		-0.20	(-0.73, 0.34)	0.47		0.55	(0.15, 0.95)	0.008		0.64	(0.24, 1.04)	0.002		0.62	(0.25, 1.00)	0.001
Asian		-0.63	(-0.83, -0.43)	<0.001		0.02	(-0.14, 0.17)	0.84		0.06	(-0.10, 0.21)	0.48		0.02	(-0.13, 0.16)	0.84
Black		-1.08	(-1.28, -0.89)	<0.001		-0.48	(-0.62, -0.33)	<0.001		-0.20	(-0.35, -0.06)	0.007		-0.29	(-0.42, -0.15)	<0.001
Mixed/Others		-0.30	(-0.51, -0.09)	0.005		-0.16	(-0.32, -0.002)	0.05		-0.12	(-0.28, 0.04)	0.14		-0.13	(-0.27, 0.02)	0.09
Smoking status																
Never		Ref				Ref				Ref				Ref		
Previous		-0.03	(-0.10, 0.03)	0.33		-0.02	(-0.07, 0.03)	0.51		-0.02	(-0.07, 0.03)	0.40		-0.03	(-0.07, 0.02)	0.29
Current		-0.23	(-0.33, -0.12)	<0.001		-0.22	(-0.30, -0.14)	<0.001		-0.19	(-0.27, -0.11)	<0.001		-0.20	(-0.27, -0.12)	<0.001
SBP (per 10mmHg)	-0.15	-0.02	(-0.04, -0.01)	0.01	-0.18	-0.02	(-0.03, -0.01)	0.003	-0.25	-0.03	(-0.04, -0.01)	<0.001	-0.26	-0.03	(-0.04, -0.01)	<0.001
Refraction (per 1D)	0.003	0.005	(-0.01, 0.03)	0.60	0.002	0.003	(-0.01, 0.02)	0.66	-0.15	-0.21	(-0.22, -0.19)	<0.001	-0.12	-0.15	(-0.17, -0.14)	<0.001
IOP_{cc} (mmHg)	0.01	0.01	(0.001, 0.03)	0.03	0.002	0.001	(-0.007, 0.01)	0.75	0.001	0.001	(-0.008, 0.01)	0.85	0.002	0.001	(-0.007, 0.01)	0.77
Corneal hysteresis	-0.01	-0.02	(-0.03, 0.003)	0.10	-0.006	-0.006	(-0.02, 0.007)	0.37	0.011	0.01	(-0.001, 0.03)	0.08	0.007	0.007	(-0.006, 0.02)	0.29

Adjusted for age, gender, ethnicity, Townsend deprivation index, height, smoking status, systolic blood pressure, refraction, IOP_{cc}, and corneal hysteresis

β = standardised beta; *B*= unstandardised beta; P= P value; SBP= systolic blood pressure; IOP_{cc}= Corneal-compensated intraocular pressure

Table S3. Multivariate analysis of demographics and risk factors with the thickness of ISOS-RPE layer

	Central subfield				Average Inner Subfield				Average Outer Subfield				Total Average			
	β	*B*	95% CI	P	β	*B*	95% CI	P	β	B	95% CI	P	β	*B*	95% CI	P
Age	-0.06	-0.05	(-0.07, -0.04)	<0.001	0.003	0.002	(-0.006, 0.01)	0.62	0.01	0.006	(-0.001, 0.01)	0.09	0.006	0.003	(-0.004, 0.01)	0.36
Sex																
Male		Ref				Ref				Ref				Ref		
Female		0.54	(0.31, 0.77)	<0.001		0.04	(-0.14, 0.22)	0.64		-0.02	(-0.16, 0.12)	0.75		-0.006	(-0.15, 0.14)	0.94
Race																
White		Ref				Ref				Ref				Ref		
Chinese		0.97	(-0.39, 2.32)	0.16		0.81	(-0.26, 1.87)	0.14		0.17	(-0.66, 1.01)	0.69		0.40	(-0.47, 1.27)	0.37
Asian		0.36	(-0.15, 0.88)	0.17		0.27	(-0.13, 0.67)	0.19		-0.15	(-0.47, 0.16)	0.33		-0.05	(-0.38, 0.28)	0.76
Black		-2.97	(-3.47, -2.48)	<0.001		-3.03	(-3.42, -2.64)	<0.001		-2.43	(-2.73, -2.12)	<0.001		-2.55	(-2.87, -2.23)	<0.001
Mixed/Others		-1.04	(-1.57, -0.51)	<0.001		-1.07	(-1.48, -0.65)	<0.001		-0.92	(-1.24, -0.59)	<0.001		-0.93	(-1.27, -0.59)	<0.001
Smoking status																
Never		Ref				Ref				Ref				Ref		
Previous		0.32	(0.15, 0.49)	<0.001		0.17	(0.03, 0.30)	0.02		0.06	(-0.05, 0.16)	0.27		0.09	(-0.02, 0.20)	0.103
Current		0.03	(-0.25, 0.30)	0.85		-0.17	(-0.39, 0.04)	0.12		-0.35	(-0.52, -0.18)	<0.001		-0.31	(-0.48, -0.13)	0.001
SBP (per 10mmHg)	-0.11	-0.04	(-0.08, 0.005)	0.08	-0.04	-0.01	(-0.05, 0.02)	0.53	-0.14	-0.03	(-0.06, -0.01)	0.02	-0.12	-0.03	(-0.06, 0.0003)	0.052
Refraction (per 1D)	0.02	0.08	(0.03, 0.13)	0.002	0.001	0.004	(-0.04, 0.04)	0.83	0.01	0.03	(0.0004, 0.06)	0.05	0.01	0.03	(-0.003, 0.06)	0.079
IOP_{cc} (mmHg)	0.03	0.07	(0.04, 0.10)	<0.001	0.02	0.05	(0.02, 0.07)	<0.001	0.03	0.04	(0.02, 0.06)	<0.001	0.03	0.04	(0.02, 0.06)	<0.001
Corneal hysteresis	0.02	0.07	(0.02, 0.12)	0.004	0.02	0.06	(0.02, 0.10)	0.001	0.02	0.04	(0.009, 0.07)	0.01	0.02	0.04	(0.01, 0.07)	0.005

Adjusted for age, gender, ethnicity, Townsend deprivation index, height, smoking status, systolic blood pressure, refraction, IOP_{cc}, and corneal hysteresis
β = standardised beta; *B*= unstandardised beta; P= P value; SBP= systolic blood pressure; IOP_{cc}= Corneal-compensated intraocular pressure
